# Land cover affects microclimate and temperature suitability for arbovirus transmission in an urban landscape

**DOI:** 10.1371/journal.pntd.0008614

**Published:** 2020-09-21

**Authors:** Michael C. Wimberly, Justin K. Davis, Michelle V. Evans, Andrea Hess, Philip M. Newberry, Nicole Solano-Asamoah, Courtney C. Murdock

**Affiliations:** 1 Department of Geography and Environmental Suitability, University of Oklahoma, Norman Oklahoma, United States of America; 2 Odum School of Ecology, University of Georgia, Athens, Georgia, United States of America; 3 Center for Ecology of Infectious Diseases, University of Georgia, Athens, Georgia, United States of America; 4 Department of Infectious Diseases, University of Georgia, Athens, Georgia, United States of America; 5 Center for Tropical Global and Emerging Diseases, University of Georgia, Athens, Georgia, United States of America; 6 Center for Vaccines and Immunology, University of Georgia, Athens, Georgia, United States of America; 7 River Basin Center, University of Georgia, Athens, Georgia, United States of America; 8 Department of Entomology, College of Agriculture and Life Sciences, Cornell University, Ithaca, New York, United States of America; Universita degli Studi di Pavia, ITALY

## Abstract

The emergence of mosquito-transmitted viruses poses a global threat to human health. Combining mechanistic epidemiological models based on temperature-trait relationships with climatological data is a powerful technique for environmental risk assessment. However, a limitation of this approach is that the local microclimates experienced by mosquitoes can differ substantially from macroclimate measurements, particularly in heterogeneous urban environments. To address this scaling mismatch, we modeled spatial variation in microclimate temperatures and the thermal potential for dengue transmission by *Aedes albopictus* across an urban-to-rural gradient in Athens-Clarke County GA. Microclimate data were collected across gradients of tree cover and impervious surface cover. We developed statistical models to predict daily minimum and maximum microclimate temperatures using coarse-resolution gridded macroclimate data (4000 m) and high-resolution land cover data (30 m). The resulting high-resolution microclimate maps were integrated with temperature-dependent mosquito abundance and vectorial capacity models to generate monthly predictions for the summer and early fall of 2018. The highest vectorial capacities were predicted for patches of trees in urban areas with high cover of impervious surfaces. Vectorial capacity was most sensitive to tree cover during the summer and became more sensitive to impervious surfaces in the early fall. Predictions from the same models using temperature data from a local meteorological station consistently over-predicted vectorial capacity compared to the microclimate-based estimates. This work demonstrates that it is feasible to model variation in mosquito microenvironments across an urban-to-rural gradient using satellite Earth observations. Epidemiological models applied to the microclimate maps revealed localized patterns of temperature suitability for disease transmission that would not be detectable using macroclimate data. Incorporating microclimate data into disease transmission models has the potential to yield more spatially precise and ecologically interpretable metrics of mosquito-borne disease transmission risk in urban landscapes.

## Author summary

Predicting the effects of temperature on mosquito abundance and arbovirus transmission cycles is essential for mapping hot spots of disease risk and projecting responses to climate change. In urban landscapes, the built environment and natural features create distinctive environments. Buildings and roads generate warmer conditions through the urban heat island effect, while vegetation can have a cooling effect because of shading and evaporative heat loss. We used land cover data to map microclimate temperature in Athens-Clarke County, GA and applied a temperature-dependent vectorial capacity model to predict the effects of microclimate on dengue transmission by *Aedes albopictus*. The highest vectorial capacity was predicted in patches of trees located in the urbanized portion of the study area. These locations had relatively warm nighttime and cool daytime temperature, which kept temperatures close to the optimum for disease transmission. This work demonstrates the feasibility of predicting variation in mosquito microenvironments in urban landscapes using satellite Earth observations. Incorporating microclimate data into disease transmission models has the potential to yield more spatially precise and ecologically interpretable metrics of mosquito-borne disease transmission risk.

## Introduction

Mosquito-transmitted arboviruses are a human health threat of worldwide significance. Given highly interconnected global transportation networks combined with rising temperatures associated with climate change and increasing levels of urbanization, there is concern that arbovirus outbreaks will happen with greater frequency in cities throughout the world [1–4]. Understanding when and where such outbreaks are most likely to occur will be essential to support disease prevention and control efforts. Accurate arbovirus risk maps can be used to target surveillance in locations where environmental conditions are conducive to disease transmission [5, 6] and to direct mosquito control in response to arbovirus outbreaks [7]. To be more useful for public health applications in cities, these maps need to capture the neighborhood-level effects of key environmental factors and predict the resulting variation in disease risk across heterogeneous urban landscapes. The present study addresses this need by modeling spatial variation in temperature across an urban-to-rural gradient in the southeastern United States and applying an epidemiological model to determine how these environmental patterns influence the potential for dengue transmission by *Aedes albopictus* (Skuse).

Meteorological factors such as temperature, precipitation, and humidity are known to have strong influences on mosquito population dynamics and disease transmission cycles [8, 9]. These relationships provide the basis for climate-driven mosquito range and disease risk maps, which have been developed using rule-based predictions [10], empirical pattern-matching approaches [11–14], and mechanistic models that capture climate influences on critical biological traits [15–18]. Model predictions are typically based on broad-scale macroclimate data from weather stations or coarse-grained meteorological grids. However, combining individual-level models of mosquito-environment relationships with macroclimate data leads to scaling errors when predictions are extrapolated across space and time [19, 20]. There is growing evidence that temperature measurements from widely-used macroclimate datasets are often decoupled from the thermal tolerances of species that occupy distinctive habitats with characteristic microclimates [21, 22]. Thermal microclimate refugia influenced by vegetation and topography can allow species to persist in areas where broader macroclimate conditions appear to be unsuitable. Progress has been made in identifying and mapping these refugia by integrating data from networks of inexpensive microclimate sensors with geospatial datasets characterizing topography and land cover [23–25].

Environmental heterogeneity is particularly high in cities, where variation in physical and social environments is known to influence the abundance and quality of larval habitats, availability of zoonotic hosts, and human exposure to mosquitoes [26, 27]. This fine-grained environmental variation influences patterns of mosquito abundance and disease transmission at the level of individual neighborhoods and city blocks [28–30]. The built environment also exerts strong influences on local microclimates [31], and understanding the effects of urban temperature variations on mosquito life cycles and transmission processes has been highlighted as an important research direction [32]. In most studies to date, the effects of habitat on mosquitoes have been modeled using land cover, land use, and socioeconomic indicators as indirect proxies [33–35]. However, recent studies combining direct monitoring of urban microclimates with field experiments [36] and observations of mosquito abundance [37] have found that variation in local temperatures account for some of the differences in mosquito abundance and life history traits across urban, suburban, and rural habitats. These results highlight the prospects for synthesizing our understanding of the thermal biology of mosquitoes with knowledge of the physical dynamics of urban climate to determine when and where microclimate conditions for disease transmission will be highest.

The urban environment influences microclimates through a variety of interactions with solar radiation, moisture, and wind speed [38]. An important factor is the prevalence of impervious surfaces such as buildings, roads, and parking lots, which absorb heat more rapidly during the day and release it more slowly at night compared to vegetated surfaces. As a result, land surface temperatures [39] and near-surface air temperatures [40] are typically highest in urban centers dominated by impervious surfaces. This urban heat island effect tends to be more pronounced at night than during the day. In contrast, tree cover reduces near-surface air temperatures as a result of shading and latent heat loss through evapotranspiration [41]. The interactions between land cover and temperature have been well documented in many cities, and have been applied to develop predictive models of urban microclimates. The resulting maps of urban heat islands have been used for public health assessments, mainly focusing on the risks associated with extreme temperatures [42]. To our knowledge, no studies have mapped mosquito-relevant microclimates in an urban setting to explore impacts on the spatial patterns of disease transmission potential.

At present, our ability to understand the climatic determinants of arbovirus transmission and predict the locations of highest risk within urban settings remains limited due to the general misalignment of scales at which meteorological data are collected versus the scales at which mosquitoes interact with the environment. To address this gap, we developed an empirical model of microclimate and land cover in Athens, GA and linked the results with a temperature-dependent epidemiological model of dengue transmission by the invasive mosquito *Ae*. *albopictus*. This species has been a primary vector of outbreaks of chikungunya and dengue viruses in tropical regions [43–45] as well as temperate locations in Italy [46], France [47], and Japan [48]. The goal of our study was to assess the climatic suitability for dengue transmission by *Ae*. *albopictus* in a temperate region of the southeastern United States, with a focus on fine-scale spatial variation along an urban-to-rural gradient. Our specific objectives were to: (1) Determine the influences of tree cover and impervious surface on daily minimum and maximum temperatures, (2) Develop a predictive spatial model to map daily microclimate at a fine-grained (30 m) spatial resolution, (3), Use a temperature-dependent model of vectorial capacity to determine how microclimate temperature influences spatial patterns of dengue transmission potential by *Ae*. *albopictus* and how these patterns change throughout the transmission season, and (4) Determine where high thermal suitability for dengue transmission occurs in locations with high human population density.

## Methods

### Study area

**Fig 1:**
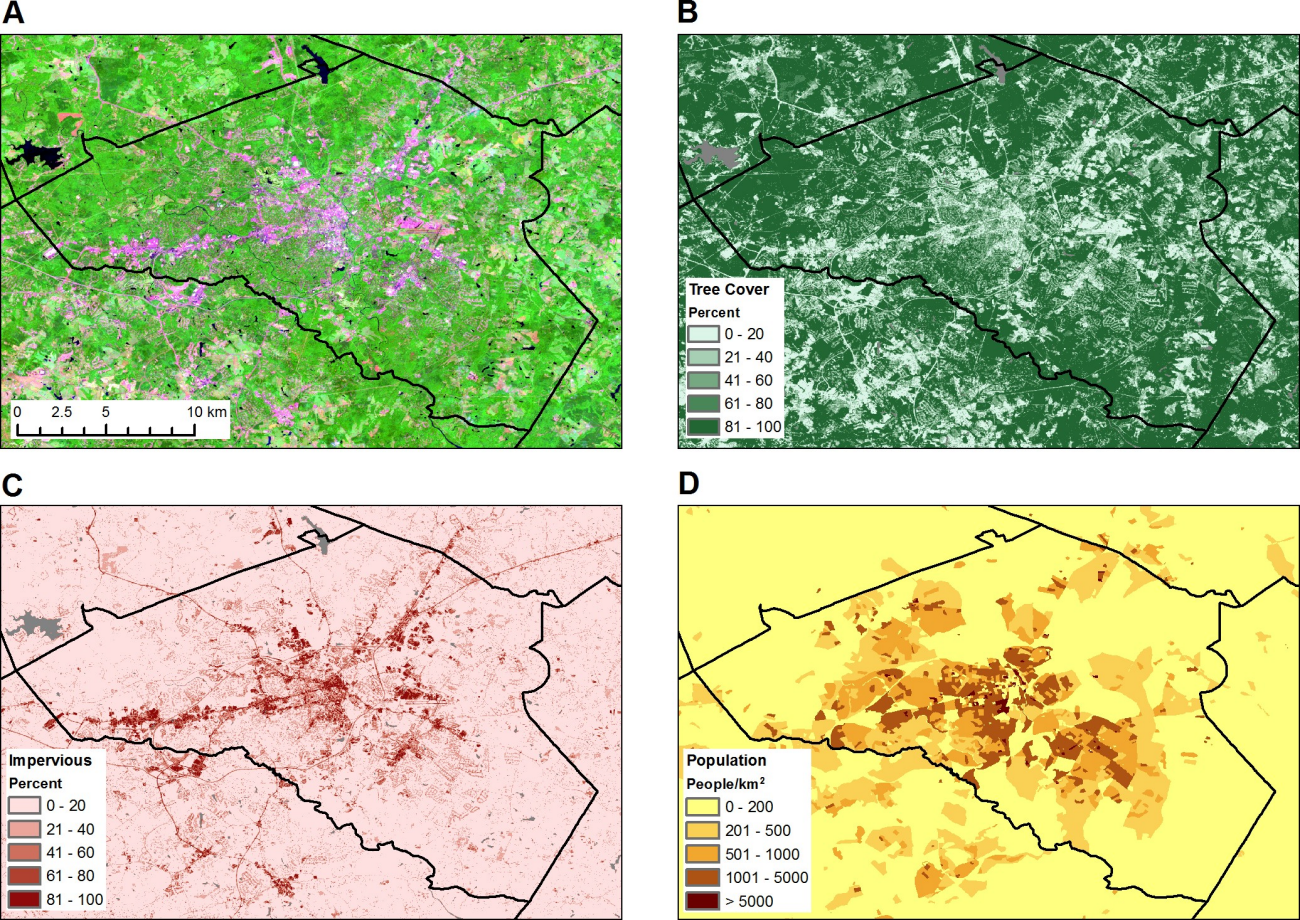
Maps of the Athens-Clarke County, GA study area. A) False-color Sentinel-2 image from 2017 with the shortwave infrared band displayed as red, the near infrared band displayed as green, and the green band displayed as blue. With this band combination, tree-covered areas are green and impervious surfaces are pink and red.; B) Tree cover map from Sentinel-2 imagery with water bodies displayed as gray; C) Impervious surface map from Sentinel-2 imagery with water bodies displayed as gray; D) Block-level population density map from the 2010 U.S. Census. Solid black lines represent county boundaries. The maps were produced using ArcGIS version 10.6.

Our study area was Athens-Clarke County Georgia, USA, which encompasses 313 km^2^ with a total population of 127,330 in 2018 (Fig 1). The urban center of downtown Athens, GA and the University of Georgia (UGA) campus are located in the middle of the study area, with high-density development extending along the major highway corridors that connect to neighboring counties. These developed areas are surrounded by residential suburbs, particularly in the western and southeastern portions of the county. The outskirts of the county are mainly rural with a mix of low-density subdivisions, pasture, and forests. Climate is subtropical with hot, humid summers (average July high 33°C) and mild winters (average January low 0.6°C). The average annual rainfall is 1200 mm.

### Scientific workflow

**Fig 2:**
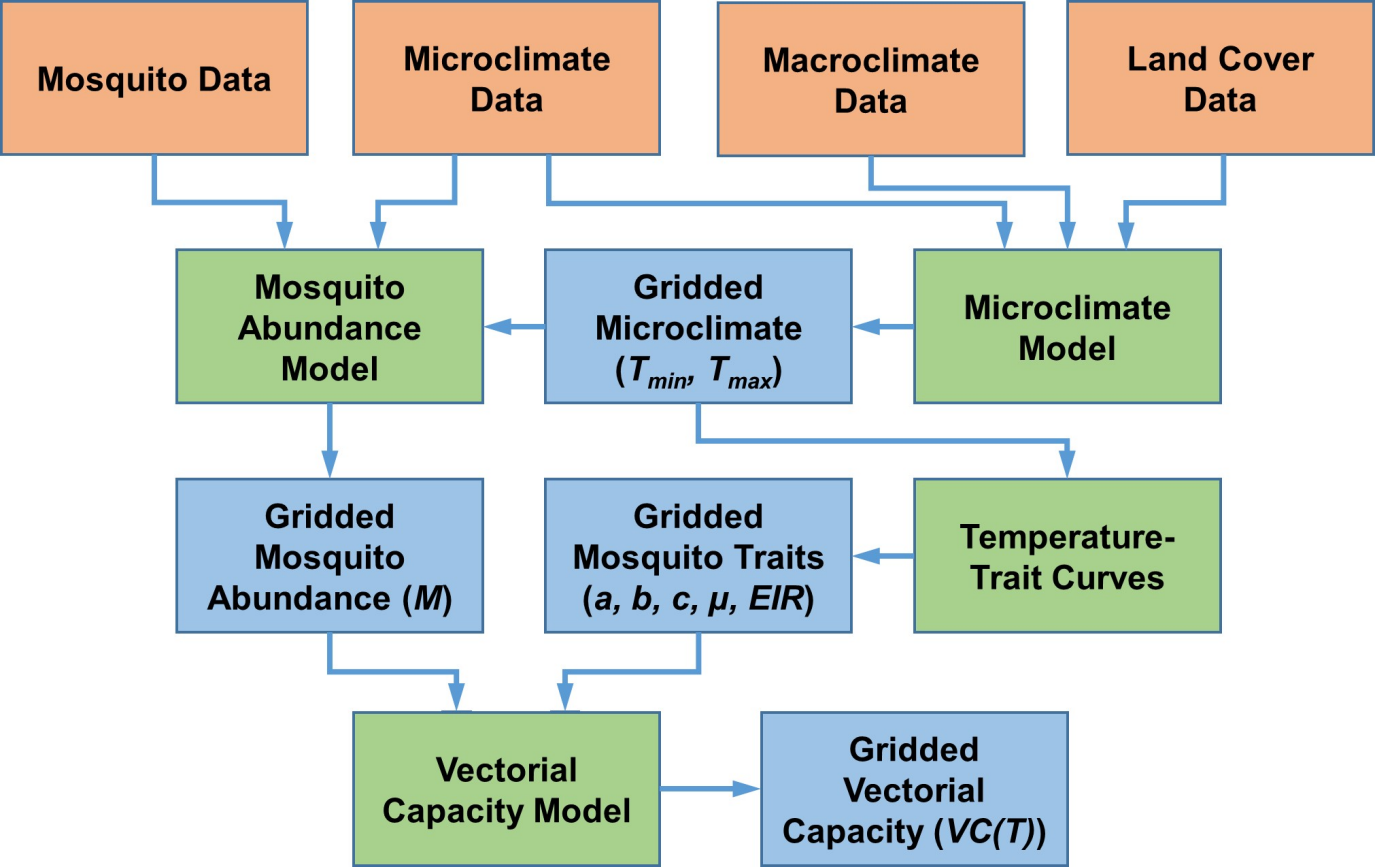
Analytical workflow for spatial modeling of microclimate temperatures and thermal potential for dengue transmission by *Aedes albopictus*. Orange boxes represent input datasets, green boxes represent models, and blue boxes represent model predictions.

The analytical workflow is presented in Fig 2. Raw data included mosquito abundance and microclimate data collected by the authors, gridded macroclimate data obtained from online archives, and a classified land cover dataset generated by the authors. The mosquito and microclimate data were used to fit a statistical model of mosquito density. The microclimate, macroclimate, and land cover data were combined to fit an empirical microclimate model and predict daily microclimate on a 30 m grid. These gridded microclimate data were used as inputs for the mosquito density model to map mosquito abundance and with published temperature-trait curves to map mosquito life history traits. The mosquito abundance and mosquito trait maps were then used as inputs for a vectorial capacity model to generate gridded estimates of temperature-dependent vectorial capacity. All data processing and modeling steps were carried out using the R language and environment for statistical computing [49].

### Microclimate sampling

We collected microclimate data from 54 locations between June 15-October 10, 2018 using temperature and relative humidity data loggers (RFID Track-it, Monarch Instruments) with radiation shields [50, 51]. These loggers have a typical accuracy of ± 0.5°C. Data were collected at nine sites, which were previously selected to cover a rural-to-urban gradient from low to high impervious surface cover in Athens [36]. At each site, six loggers were distributed within a 100 m radius plot, with individual logger locations chosen to encompass the range of tree canopy cover within the plot. The loggers were placed approximately 1 m above the ground in vegetation considered to provide suitable resting habitat for *Ae*. *albopictus*. The loggers were programmed to collect data at 10-minute intervals.

**Fig 3:**
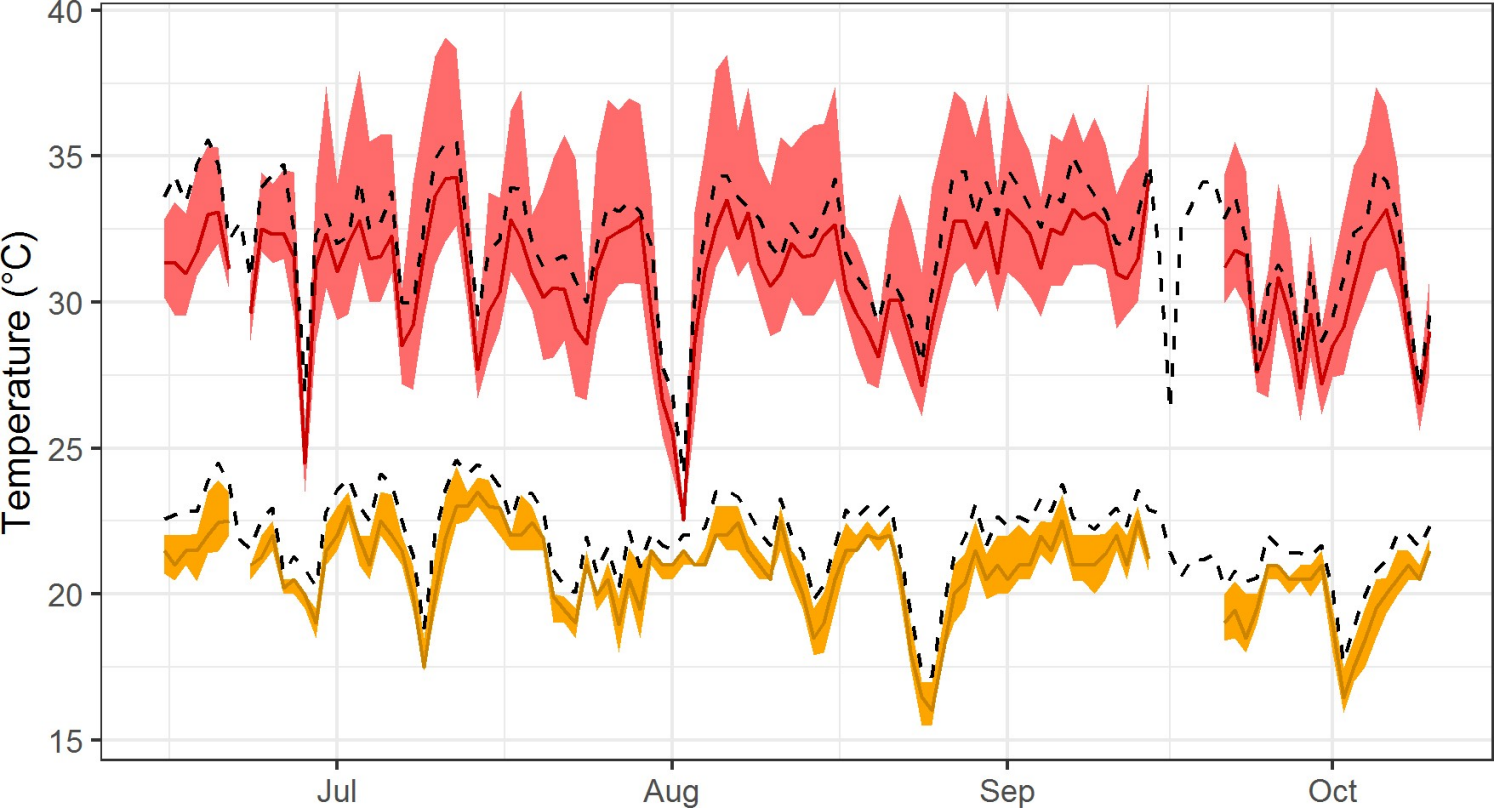
Time series of daily temperature in Athens, GA during 2018. Colored lines represent medians and shaded areas represent the 5%-95% quantile range of microclimate observations for daily minimum temperature (orange) and daily maximum temperature (red). Dashed black lines represent data collected at the University of Georgia weather station.

Data from three of the loggers were not used because of equipment malfunctions, and data were not available on June 22 and September 15–20. In total, we obtained 790,016 temperature measurements from 51 loggers over a 118-day period. The available microclimate data were used to compute daily minimum and maximum temperature for each logger (Fig 3).

### Temperature downscaling

We mapped microclimates using statistical downscaling, which is based on empirical relationships with broad-scale meteorological patterns and fine-grained data on features that influence local climate characteristics [52–54]. Sources of meteorological data include a variety of gridded datasets, which combine interpolated weather station data with other sources of geospatial information to generate meteorological surfaces that vary smoothly over tens to hundreds of kilometers. Finer-grained data relevant to local microclimate include vegetation, impervious surface, soils, and topography that vary over tens to hundreds of meters.

We modeled microclimate temperature using a linear model of the form

$$T_{id}=\alpha +\sum_{j=1}^{p}\beta_{j}C_{idj}+\sum_{k=1}^{q}\gamma_{k}L_{ik}$$ (1)

where *T*_*id*_ is the microclimate variable (minimum or maximum temperature) for a 30 m grid cell at location *i* on day *d*, the *C*_*idj*_ are *p* daily macroclimate variables indexed as *j* and measured for location *i* on day *d*, *L*_*ik*_ are *q* land cover variables indexed as *k* and measured for location *i*, *α* is the intercept parameter, *β*_*j*_ are the slope parameters for the macroclimate variables, and *γ*_*k*_ are the slope parameters for the land cover variables.

We used macroclimate data from the GridMET dataset, which provides daily meteorological data for 4 km grid cells [55]. GridMET combines hourly meteorological data from the North American Land Data Assimilation System Phase 2 [NLDAS-2, 56] with higher resolution climatological data from the Parameter-Elevation Regression on Independent Slopes Model [PRISM, 57]. Macroclimate variables included minimum and maximum temperature, minimum and maximum relative humidity, wind velocity, solar radiation, and precipitation (Table 1). The macroclimate data were rescaled to a 30 m resolution using bilinear interpolation for overlay with the land cover variables.

**Table 1:** Predictor variables considered in the development of linear models to predict microclimate temperatures based on macroclimate and land cover.

Variable Type	Variable Code	Description	Units
Macroclimate	TN	Minimum temperature	°C
	TX	Maximum temperature	°C
	RHN	Minimum relative humidity	%
	RHX	Maximum relative humidity	%
	WIND	Wind speed	m/s
	SRAD	Total solar radiation	W/m^2^
	PREC	Total precipitation	mm
Land Cover	TREE	Percent tree cover	%
	TREE3	Percent tree cover (3 x 3 pixel focal mean)	%
	TREE5	Percent tree cover (5 x 5 pixel focal mean)	%
	IMPERV	Impervious cover	%
	IMPERV3	Impervious cover (3 x 3 pixel focal mean)	%
	IMPERV5	Impervious cover (5 x 5 pixel focal mean)	%
	IMPERV1K	Impervious cover (1 km radius focal mean)	%
	IMPERV2K	Impervious cover (2 km radius focal mean)	%

Custom 2018 land cover data were mapped for Athens-Clarke County at a 10 m resolution by classifying Sentinel-1 synthetic aperture radar imagery and Sentinel-2 optical-infrared imagery. We mapped percent impervious surface cover and percent tree cover because of their known associations with urban microclimates. Details of the remote sensing methods and accuracy assessment results are available in the supporting information (S1 File). The 10 m tree cover pixels were aggregated to 30 m pixels to measure local effects of shading on microclimate. We then calculated mean tree cover within 3 x 3 and 5 x 5 pixel focal windows to capture potential cumulative effects across broader areas. Impervious surface was similarly aggregated to 30 m pixels and summarized as a mean for 3 x 3 and 5 x 5 focal windows. We also summarized impervious surface using larger circular windows of 1 km and 2 km. Our goal with these larger focal windows was to capture urban heat island effects arising from the broader-scale effects of increased thermal storage and re-radiation of heat in urbanized areas.

We compared multiple models with different combinations of macroclimate and land cover variables as predictors. Initial models included at least one macroclimate temperature variable (either minimum or maximum temperature) and two land cover variables (one tree cover variable and one impervious surface variable). All possible combinations of these variables were explored to determine the best-fitting model, and interactions between tree cover and impervious surface were also considered. After determining the initial models, we used a forward stepwise approach to identify additional macroclimate and land cover variables that improved model fit. Model fit was assessed using Akaike’s information criterion (AIC). We also calculated variance inflations factors (VIFs) and excluded models with combinations of variables that resulted in high multicollinearity.

The accuracies of the final microclimate models were assessed using cross validation. For each iteration of the cross-validation algorithm, data from one logger were excluded from the model fitting data, the model was fitted with data from the remaining loggers, and model predictions were then compared to observations from the excluded logger. This procedure was repeated 51 times (once for each logger) to generate a cross-validation dataset in which all observations were compared with independent predictions.

### Vectorial capacity modeling

To infer how fine scale spatial heterogeneity in temperature influences environmental suitability and risk for arbovirus transmission, we used a temperature-dependent expression for vectorial capacity (*VC*) for dengue transmission by *Ae*. *albopictus* [36] to predict temperature suitability for transmission at the level of a 30 m grid cell. *VC* estimates the total number of infectious bites that would eventually arise from all the mosquitoes biting a single infected human on a single day [58].

VC(T)=M(T)a(T)2b(T)c(T)exp(−μ(T)EIR(T))μ(T) (2)

We used temperature-trait relationships reported previously by Mordecai et al. [17], where the per capita biting rate (*a*), the transmission (*b*) and infection (*c*) probabilities, the per capita mortality rate (*μ*) and the extrinsic incubation rate (*EIR*) were all modeled as non-linear functions of temperature (*T*). When combined in the *VC(T)* equation they provide a mechanistic estimate of the potential for disease transmission based on the rate at which mosquitoes become infectious and transmit the disease (represented in the numerator) and the lifespan of the mosquito (represented by the mortality rate in the denominator). Sensitivity analysis of these traits in a model of dengue transmission by *Ae*. *albopictus* found that across all temperatures, transmission was highly sensitive to *EIR*, which measures the rate at which infected mosquitoes become infectious [17]. At higher temperatures, transmission was most sensitive to *μ*, which measures the mortality rate of the mosquitoes.

Mosquito abundance (*M*) is also sensitive to *T* because of temperature effects on mosquito fecundity, growth, and survival. Mordecai et al. [17, 59] proposed the following mechanistic equation for *M(T)*,

$$M(T)=\frac{EFD(T)pEA(T)MDR(T)}{{\mu (T)}^{2}}$$ (3)

where eggs produced per female per day (*EFD*), egg-to-adult survival (*pEA*), and mosquito development rate (*MDR*) are all non-linear functions of *T*.

This mechanistic approach for modeling *M(T)* has been applied to generate continental-scale predictions of mosquito-borne disease transmission across broad climate gradients [17, 18, 59, 60]. To obtain locally calibrated estimates of the relationship of mosquito abundance with temperature for predicting neighborhood-level patterns in Athens-Clarke County, we used field data on *Ae*. *albopictus* abundance and microclimate temperatures to develop an empirical model of mosquito abundance. Mosquito data were collected using BG-Sentinel 2 traps that were deployed at the same nine sites used for microclimate monitoring. Adult mosquito samples were collected 26 times between May 2016 to December 2017 [37]. Traps were run continuously for 48 hours and were baited with an octenol (1-octen-3-ol) lure. At the same locations and over the same time period, microclimate temperature data were collected at 10-minute intervals using six loggers at each site. A negative binomial generalized additive model was used to predict mosquito abundance as a smoothed function of mean minimum and maximum daily temperatures averaged over the week preceding the collection date.

$$M(T_{min},T_{max})=b+s(T_{min})+s(T_{max})$$ (4)

where mean daily minimum microclimate temperature (*T*_*min*_) and mean daily maximum microclimate temperature (*T*_*min*_) were summarized over the week preceding the day of the mosquito collection. The *s(T*_*min*_*)* and *s(T*_*max*_*)* terms were smoothed functions based on thin-plate splines. The model was fit in R using the gam function from the mgcv package with default settings [49, 61]. Model predictions were cross-validated across the nine sites using the approach previously described for the microclimate models. Background information on the mosquito data and meteorological associations of mosquito abundance can be found in Evans et al. [37].

The expression for temperature-dependent *VC(T)* assumes a constant temperature to calculate the per-generation rate of increase of a pathogen in a fully susceptible population. However, environmental temperature in the field is variable, which affects the calculation and interpretation of *VC* [62]. To calculate *VC(T)*, we first used the predicted daily mean and maximum temperature values for each pixel to estimate hourly temperature using equations from [63]. The hourly temperatures were used to generate hourly estimates of the key rate parameters in Eqs 1 and 2 using the equations from Mordecai et al. [17] The hourly rates were then used to compute monthly means for each pixel. We summarized the data for four monthly periods: June/July (June 15-July 15), July/August (July 16-August 15), August/September (August 16-September 15), and September/October (September 16-October 10). The mean rates were then used to calculate *VC(T)* for each monthly period.

Because the vectorial capacity estimates are based only on temperature, they should be interpreted as a relative metric of thermal suitability for disease transmission rather than a precise estimate of the number of infectious bites. Therefore, we transformed *VC(T)* into standardized index with a [1,0] range using the following equation

$${VC(T)}_{s}=\frac{VC(T)-{VC(T)}_{min}}{{VC(T)}_{max}-{VC(T)}_{min}}$$ (5)

where *VC(T)*_*min*_ was the minimum value of *VC(T)*, and *VC(T)*_*max*_ was the maximum value. We used these standardized values to map of *VC(T)* for each of the four monthly periods. We also compared the distribution of *VC*(*T*) values for each period with an estimate of *VC(T)* calculated using temperature data from the UGA weather station.

To identify where high thermal suitability for dengue transmission occurred in locations with high human population density, we used a simple vulnerability index. The variables used in this index included the mean of *VC(T)* calculated across all four months and log-transformed block-level population from the 2010 U.S. Census. Both variables were standardized to a [0, 1] range using the method shown in Eq 5. A geometric mean with equal weights applied to each variable was used to calculate the vulnerability index as

$$VI=\sqrt{{VC(T)}_{s}∙{LOGPOP}_{s}}$$ (6)

Where *VC(T)*_*s*_ was the standardized temperature-dependent vectorial capacity and *LOGPOP*_*s*_ was the standardized logarithm of population. This index is quantitatively similar to previous approaches for mapping vulnerability to dengue transmission [64, 65], and is conceptually similar to the vector-to-host ratio proposed by Vanwambeke [66] in that it assumes vulnerability will be highest in locations where there are high levels of vectorial capacity combined with high densities of human hosts.

## Results

### Microclimate

The best microclimate model for minimum temperature included the macroclimate variables minimum temperature and maximum humidity along with the tree cover summarized within a 5 x 5 grid cell (150 x 150 m) window and impervious surface summarized in a circular window with a 1 km radius (Table 2). The best microclimate model for maximum temperature included the macroclimate variables maximum temperature, minimum humidity, and solar radiation along with tree cover summarized for a 30 m grid cell. The minimum temperature model had a cross-validated R^2^ of 0.61 and mean absolute error of 0.76°C. The maximum temperature model had a cross-validated R^2^ of 0.75 and mean absolute error of 1.72°C.

**Table 2:** Linear regression models used to predict minimum and maximum daily temperatures as a function of macroclimate and land cover variables. Variable codes are defined in Table 1.

Microclimate Model	Variable^a^	Parameter	SE	95% CI
*T*_*min*_	intercept	-1.5539	0.4185	-2.3745, -0.7335
	TN	0.7801	0.0090	0.7624, 0.7978
	RHX	0.0449	0.0031	0.0388, 0.0510
	IMPERV1K	0.0281	0.0015	0.0188, 0.0247
	TREE5	-0.0030	0.0009	-0.0049, -0.0012
*T*_*max*_	intercept	4.7898	1.0713	2.6895, 6.8901
	TX	0.9397	0.0255	0.8897, 0.9896
	RHN	-0.0507	0.0067	-0.0639, -0.0375
	SRAD	0.0027	0.0005	0.0017, 0.0038
	TREE	-0.0184	0.0013	-0.0209, -0.0160

^a^Variable codes are defined in Table 1.

**Fig 4:**
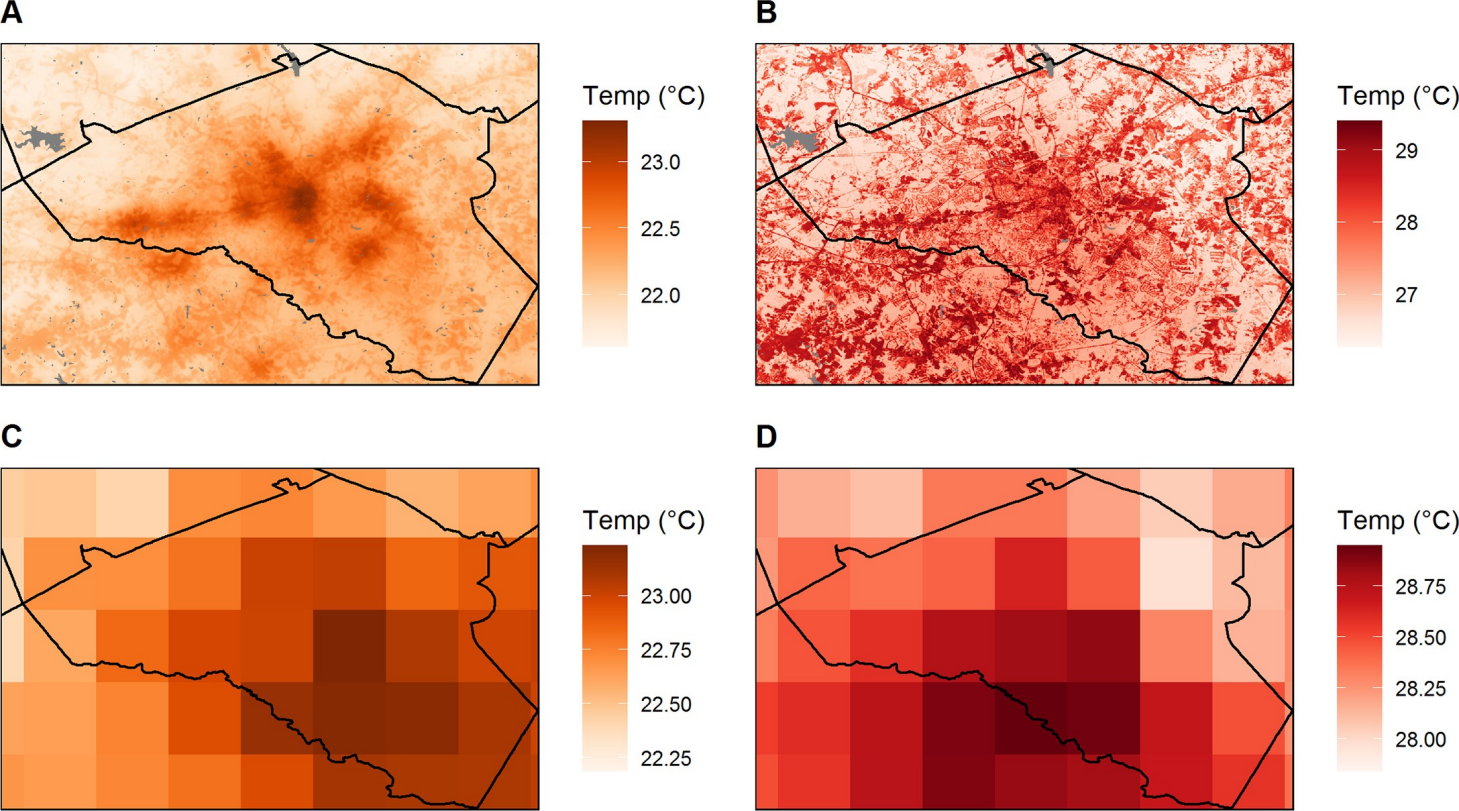
Example maps of downscaled microclimate and GridMet temperatures for July 14^th^, 2018. A) Minimum microclimate temperature. B) Maximum microclimate temperature. C) Minimum temperature from GridMET data. D) Maximum temperature from GridMET data. The maps were produced using R version 3.6.1.

The minimum temperature microclimate model predicted higher temperatures near the center of Athens, reflecting broad-scale effects of impervious surfaces through heat storage during the day and re-radiation at night (Fig 4A, Fig A in S2 File). In contrast, the maximum temperature microclimate model predicted finer-scale spatial variation due to strong local effects of tree shading during the day (Fig 4B, Fig B in S2 File). The microclimate data captured more fine-grained spatial variability than was detectable with the 4 km GridMET data (Fig 3C and 3D), highlighting the localized effects of land cover on microclimate.

### Mosquito Abundance

**Fig 5:**
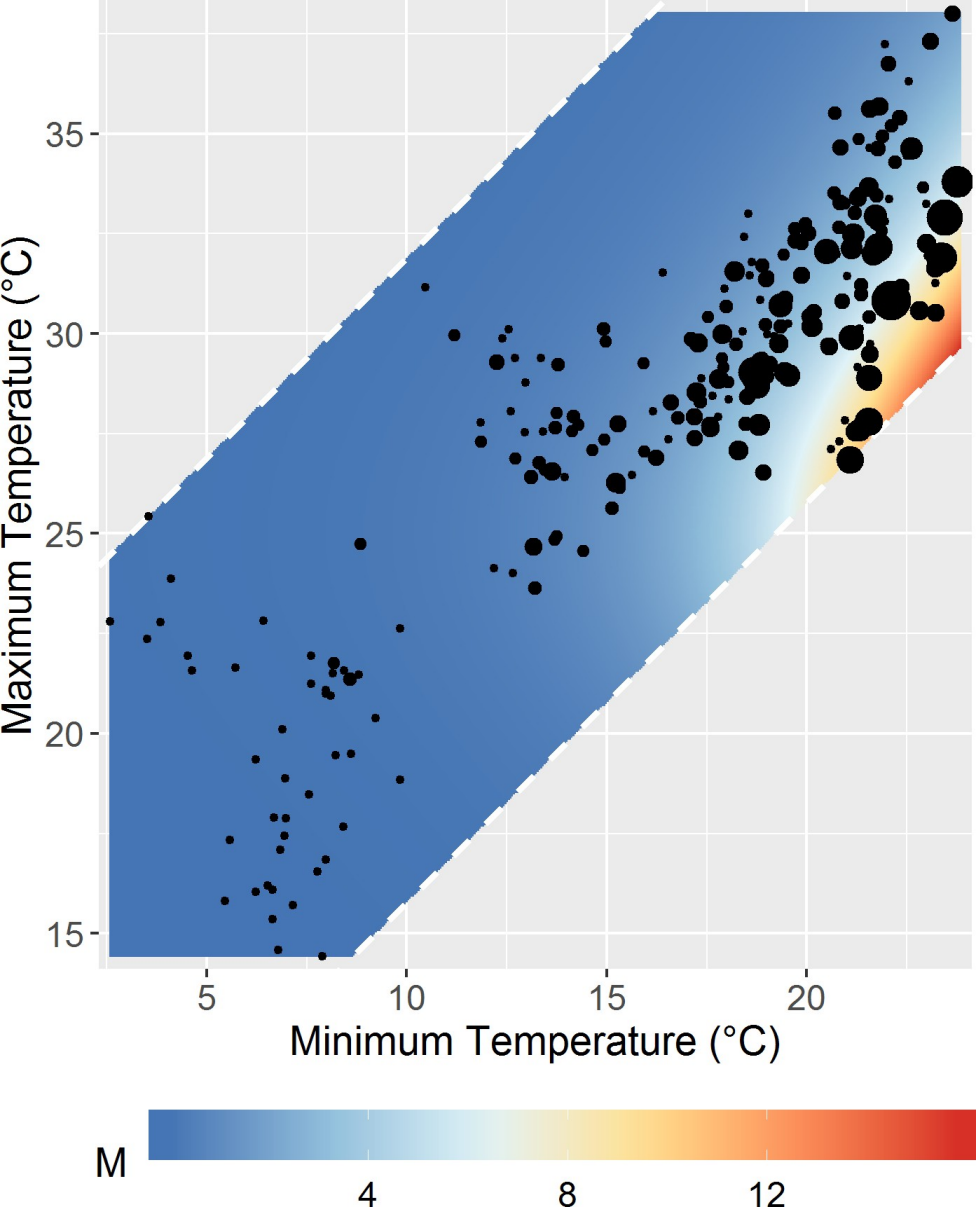
Predicted counts of *Ae*. *albopictus* abundance as a smoothed function of minimum and maximum daily temperatures summarized over the preceding week. Black dots represent observed mosquito abundance with the size of the dot proportional to the number of mosquitoes.

The empirical model of *M*(*T*_*min*_, *T*_*max*_) predicted a positive, monotonic relationship between mosquito abundance and *T*_*min*_ and a unimodal relationship between abundance and *T*_*max*_ with a peak at approximately 30°C (Fig 5). The model explained 46% percent of the deviance in mosquito abundance. The AIC of this model (1012.0) was lower than simpler models that included only minimum temperature (1028.4), only maximum temperature (1065.0), and only mean temperature (1037.9), as well as a more complex model that incorporated interactions between minimum and maximum temperature via a tensor product (1015.8). The cross-validated predictions had a mean error of 0.07, a mean absolute error of 2.3, and a median absolute error of 1.0. The Spearman rank correlation between predicted and observed values was 0.52.

When predicted monthly abundances were compared, the temperature associations of the empirical *M*(*T*_*min*_, *T*_*max*_) model (Fig C in S2 File) were different from those of the mechanistic *M(T)* model (Fig D in S2 File), with the empirical model predicting peak mosquito abundance at warmer minimum and maximum temperatures than the mechanistic model. The *VC(T)* predictions based on the empirical mosquito abundance model (Fig E in S2 File) also peaked at higher minimum temperatures than the *VC(T)* predictions based on the mechanistic model (Fig F in S2 File). The different temperature associations translated into differences in the spatial patterns of mosquito abundance (Figs H, I in S2 File) and vectorial capacity (Figs J, K in S2 File), with predictions based on the empirical mosquito abundance model tending to be lower in the outlying rural areas where temperatures were cooler.

Because the mechanistic model is a simple equilibrium model based on temperature-trait relationships determined in controlled laboratory environments, it provides generalized predictions of temperature suitability for *Ae*. *albopictus* populations. In contrast, the empirical model was directly calibrated using field and microclimate measurements, and the resulting temperature niche reflects the local influences of habitat, host availability, competition, and genetic adaptation. We expected the empirical model to provide more precise estimates of mosquito abundance within the study area, and we used it as the basis for predicting *VC(T)* and assessing temperature effects on dengue transmission potential.

### Vectorial Capacity

**Fig 6:**
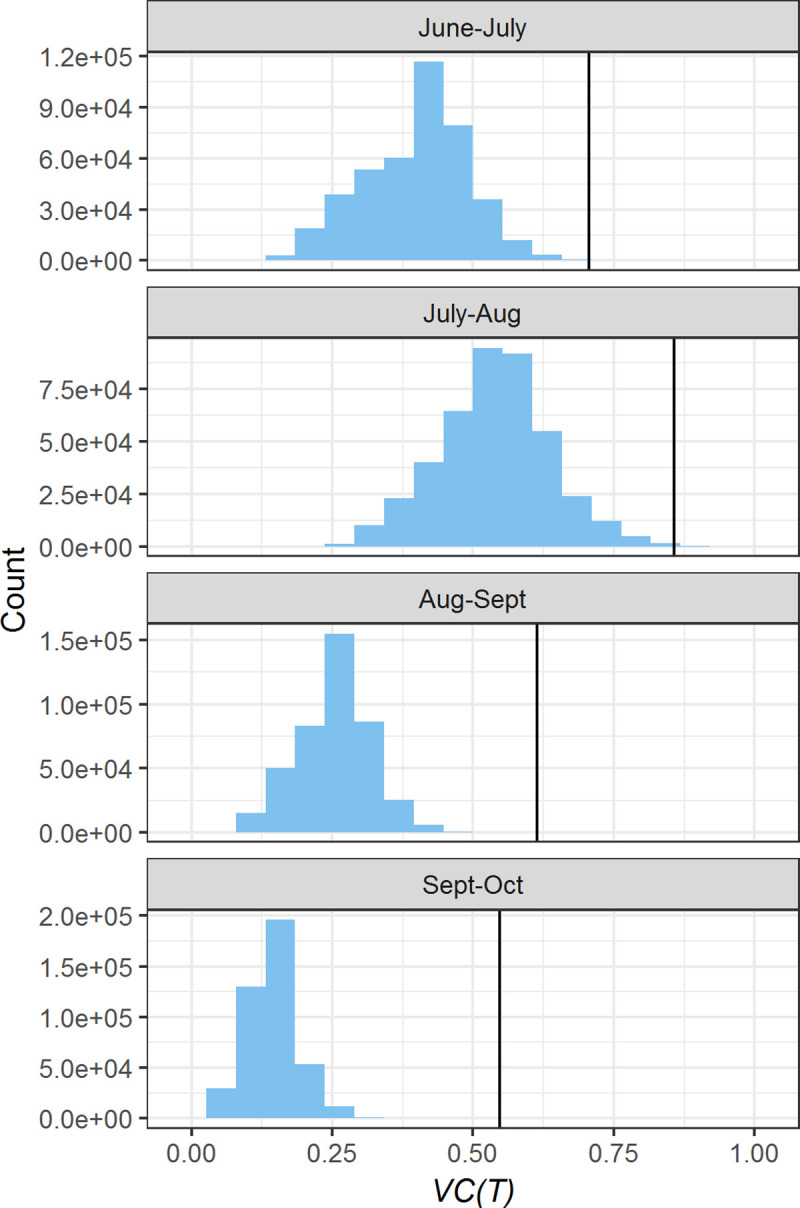
Histograms of temperature-dependent vectorial capacity (*VC(T)*) for Athens, GA during four monthly periods in 2018. Bars represent the distribution of *VC(T)* throughout the entire study area based on a sample of one million grid cells. Black lines represent point estimates of *VC(T)* based on data from the UGA weather station.

Predictions of vectorial capacity varied widely during the summer of 2018, with 99% of values falling between 4.0 and 14.2. Thus, thermal suitability for dengue transmission by *Ae*. *albopictus* was more than three times as high in the most favorable microclimates than in the least favorable microclimates. The relative *VC(T)* index increased slightly from June-July to July-Aug and then decreased in Aug-Sept and Sept-Oct (Fig 6). During all months, the *VC(T)* estimates calculated using meteorological data from the UGA weather station were higher than the distributions of *VC(T)* based on microclimate temperatures. The difference between microclimate and weather station *VC(T)* was lowest in June-July and greatest in Sept-Oct, when the weather station temperature predicted a *VC(T)* that was more than twice as high as the microclimate predictions.

**Fig 7:**
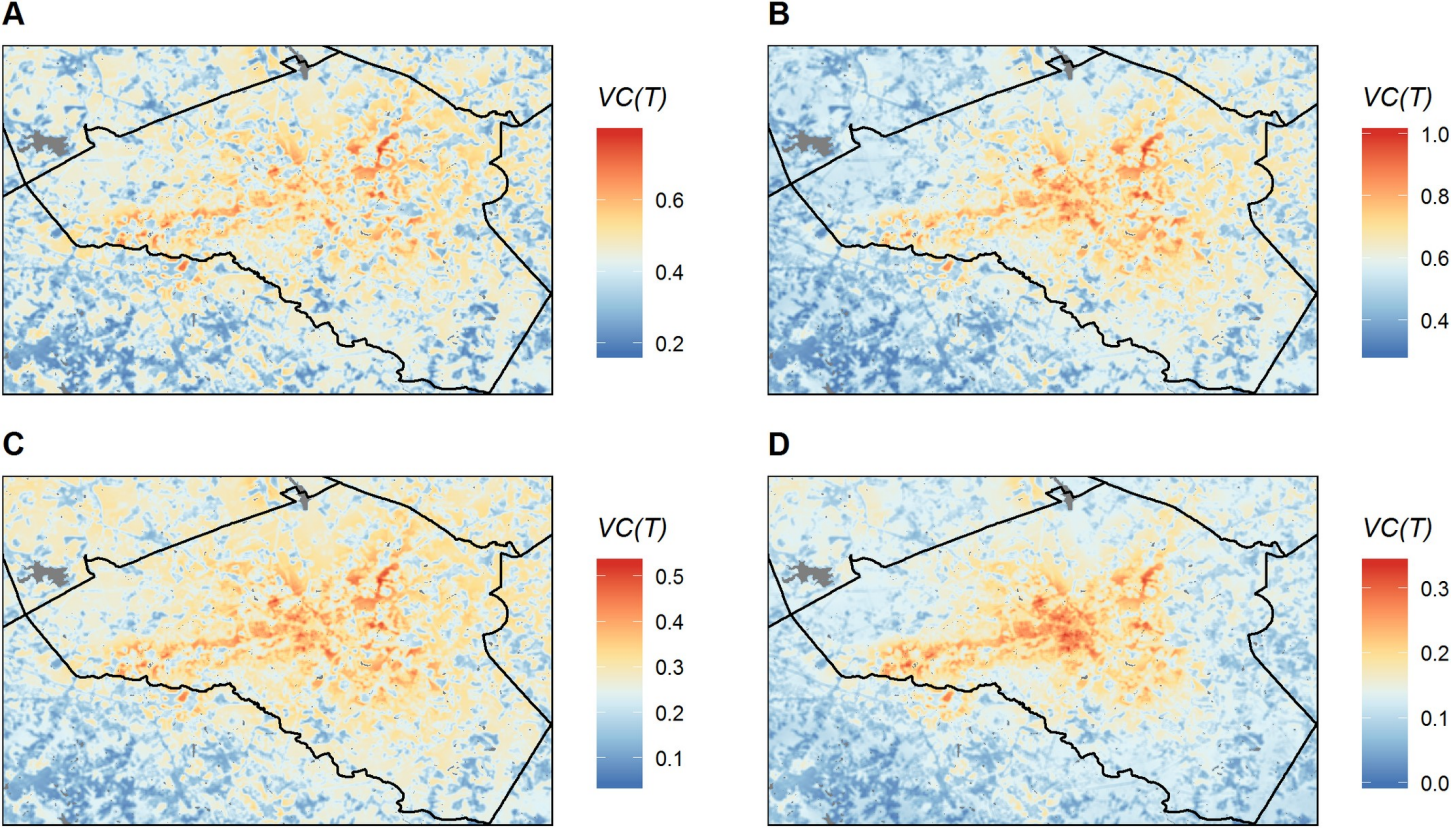
Maps of temperature-dependent vectorial capacity (*VC(T)*) for Athens-Clarke County, GA during four monthly periods in 2018. A) June-July. B) July-August. C) August-September. D) September-October. Note that a different range of *VC(T)* is represented in each map. Gray patches represent water bodies. The maps were produced using R version 3.6.1.

The spatial patterns of relative *VC(T)* in Athens-Clarke County shifted throughout the 2018 transmission season. In June-July, there were several small patches of high *VC(T)* near the center of the study area with other areas of intermediate and low *VC(T)* distributed throughout the entire county Fig 7A). Beginning in July-Aug, a larger concentration of high and intermediate *VC(T)* appeared in the middle of the study area, centered on the downtown core of Athens (Fig 7B), and this cluster of high *VC(T)* remained in Aug-Sept and Sept-Oct (Fig 7C and 7D). Patterns of *VC(T)* in Sept-Oct displayed a particularly strong urban-to-rural gradient, with areas of high *VC(T)* concentrated around downtown Athens and low *VC(T)* in most of the outlying rural areas.

**Fig 8:**
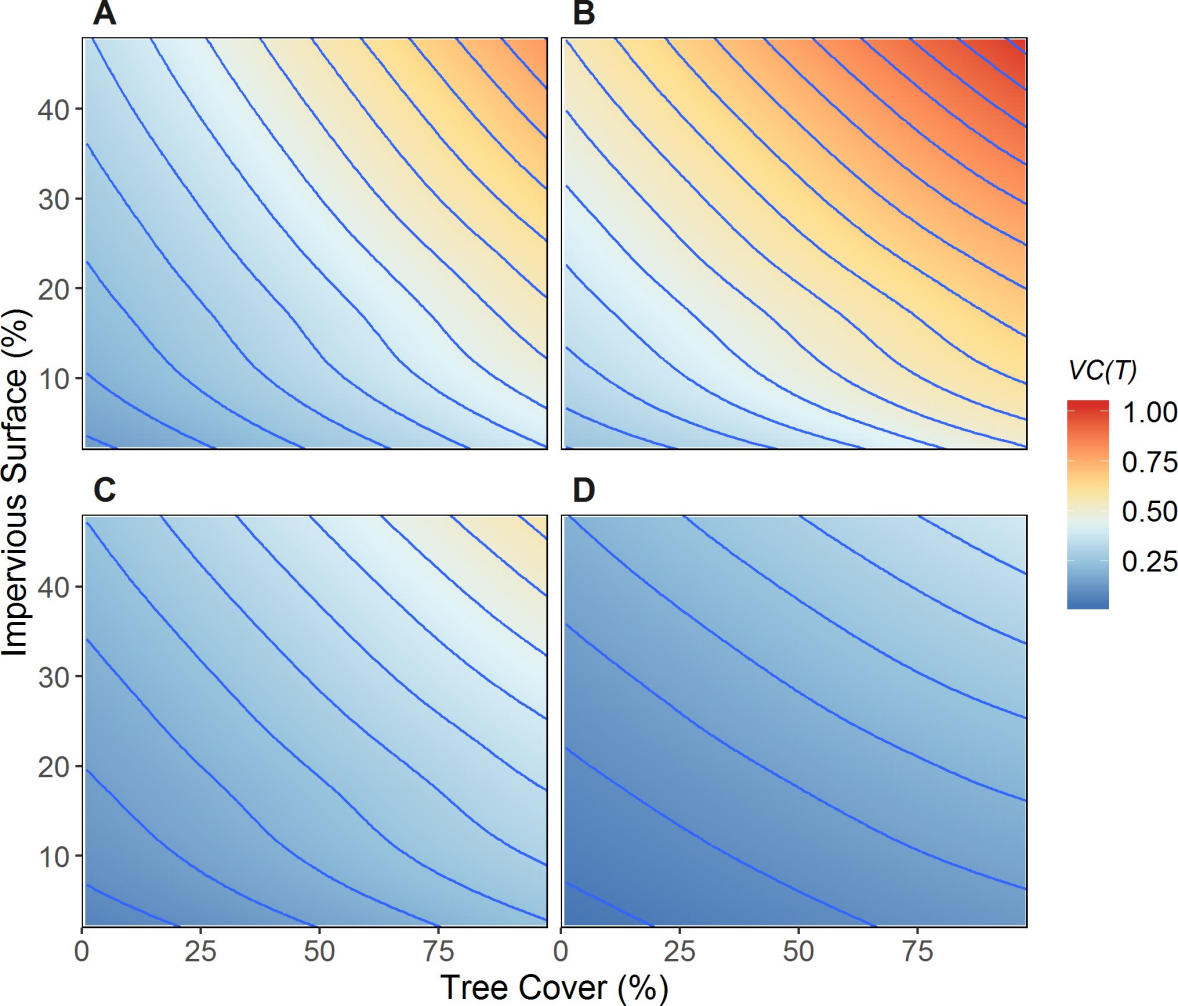
Contour plots of temperature-dependent vectorial capacity (*VC(T)*) in relation to percent tree cover (summarized locally in a 30 m grid cell) and percent impervious surface (summarized within a 1-km circular window). The response surface was generated using LOESS (localed estimated scatterplot smoothing) regression. A) June-July. B) July-August. C) August-September. D) September-October. Each blue contour line represents a one-unit change of *VC(T)*.

These shifting spatial patterns reflected the changing influences of tree cover and impervious surfaces on environmental suitability for dengue transmission throughout the season (Fig 8). In June-July, both tree cover (measured in a 30 m grid cell) and impervious surface cover (measured in a 1 km radius circular window surrounding the grid cell) were positively associated with *VC(T)*, with the highest predicted *VC(T)* in patches of high tree cover that were embedded in broader landscapes dominated by impervious surfaces (Fig 8A). There was also an interaction between the two variables, such that *VC(T)* increased more rapidly with increasing tree cover in urban landscapes with higher impervious surface cover than in more rural landscapes with low impervious surface cover. As the season progressed, the relative influence of tree cover gradually decreased while the relative influence of impervious surface cover increased. By Sept-Oct, impervious surface cover had the strongest influence on *VC(T)*, and the effects of tree cover were weaker (Fig 8D).

**Fig 9:**
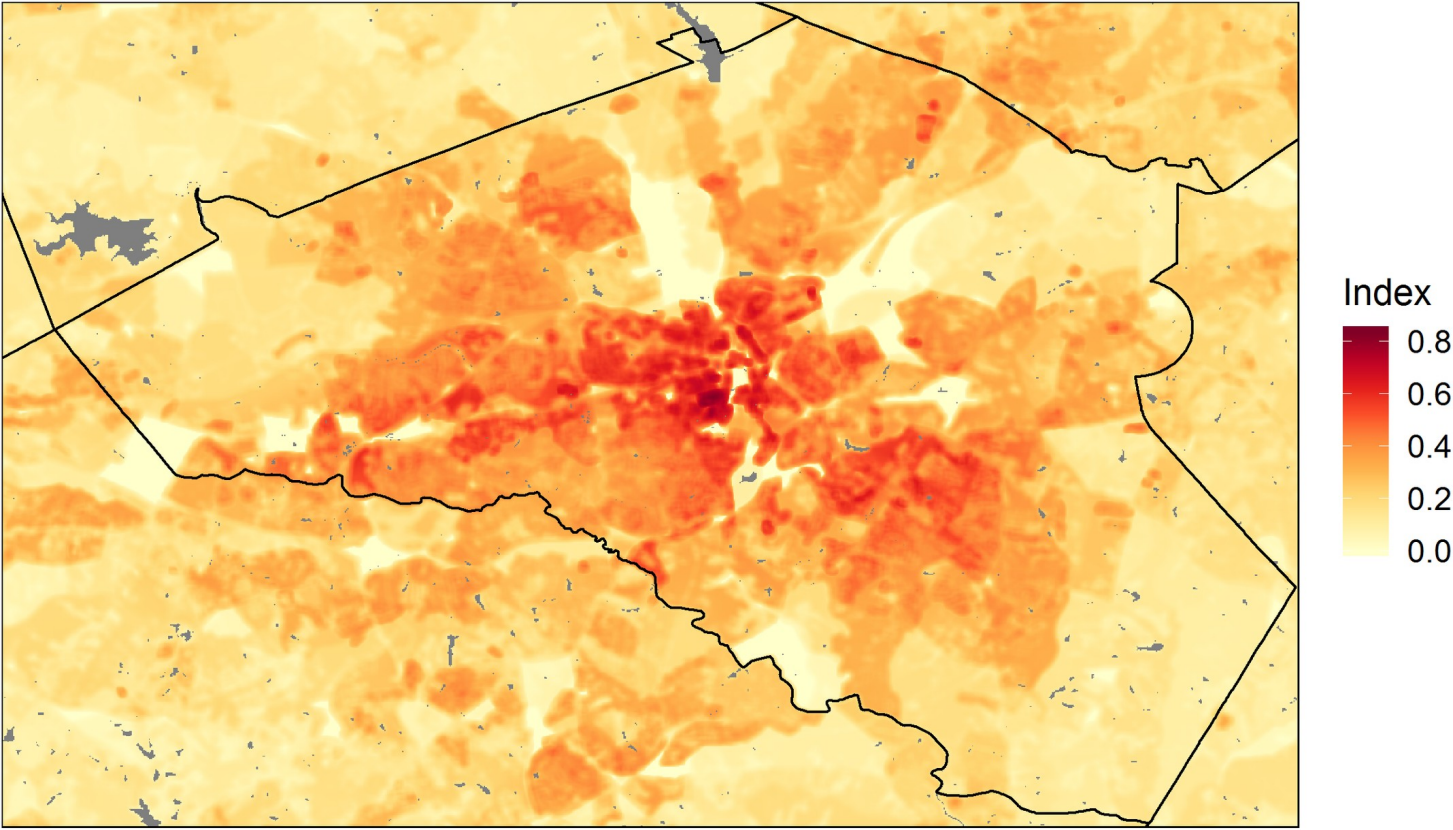
Map of a vulnerability index for dengue transmission in Athens-Clarke County, GA. The index was based on the standardized *VC(T)* for June-October 2018 and standardized, log-transformed human population density data from the U.S. Census. Gray patches represent water bodies. The map was produced using R version 3.6.1.

Although dengue is not currently transmitted locally in Athens, the vulnerability index highlighted where concentrations of people live in neighborhoods with high thermal suitability for dengue transmission (Fig 9). The areas with highest vulnerability were in older neighborhoods close to downtown Athens and in residential portions of the UGA campus, which were surrounded by high densities of impervious surfaces but also had high tree cover and had dense human populations living in closely-spaced houses, apartment buildings, and dormitories. Other areas with high vulnerability indices included dense suburban neighborhoods and clusters of apartments with high tree cover located in the southeastern portion of the study area and along the Atlanta highway in the western part of the study area.

## Discussion

The importance of temperature as an environmental determinant of mosquito life history and arbovirus transmission is well established [67]. To date, temperature-driven spatial models of arbovirus transmission have mostly been applied over relatively large geographic areas, ranging from individual countries to the entire globe, using gridded climate data. These climate datasets are derived by interpolating point data from isolated weather stations [68–70] and incorporating additional data from Earth-observing satellites [71, 72]. The resulting maps thus capture spatial variation in macroclimate at relatively coarse spatial resolutions ranging from 1–100 km per grid cell. To ensure consistency of measurements, meteorological stations are typically established at sites with relatively flat terrain and with no shading from buildings or trees [73]. In contrast, individual *Ae*. *albopictus* complete their life cycles within much smaller areas, typically covering distances of a few hundred meters or less [74]. Further, *Ae*. *albopictus* often occupies breeding and resting habitats that are shaded by vegetation canopies and near buildings. Previous research by Murdock et al. [36] has confirmed that these microclimates have distinctive temperature and humidity compared to measurements taken at meteorological stations. The present study builds on these results by showing that impervious surfaces and tree cover influence microclimate conditions, and that spatial patterns of microclimate affect both vectorial capacity and vulnerability to arbovirus transmission within the city of Athens.

An important implication of these results is that assumptions of homogeneous climate across large expanses of urban area, which are implicit in the extrapolation of data from meteorological stations and coarse-scaled macroclimate grids, can result in biased estimates of mosquito-borne disease transmission potential. In Athens, GA, predictions of *VC(T)* based on meteorological station data consistently overestimated the potential for dengue transmission compared to predictions based on microclimate data. Because of the high spatial variability of microclimate temperature regimes, locations with the highest temperature-dependent vectorial capacity occupied a relatively small portion of the county, primarily in patches of high tree cover embedded in urbanized landscapes with high impervious surface cover. Many of the locations with high vectorial capacity were located in areas with higher human population densities, giving them a disproportionate influence on human dengue risk. High spatial resolution microclimate mapping combined with spatial modeling of vectorial capacity can aid in the identification of these environmental hotspots, which can then be combined with additional information on breeding sites and human vulnerability to target surveillance and mosquito control activities.

Our spatial model of microclimate temperatures was relatively simple, but explained a substantial portion of the variation in minimum and maximum temperatures across the rural-to-urban gradient in Athens-Clarke County. We found that minimum microclimate temperatures had a strong positive association with impervious surfaces summarized within a 1-km circular window surrounding each logger, whereas maximum microclimate temperatures were negatively associated with tree cover within the 30 m pixel containing each logger. These land cover effects are consistent with the results of research in other cities and general principles of urban micrometeorology. A study of urban heat islands in Chicago (USA) found that nighttime temperatures measured within city blocks increased with impervious surface and decreased with tree cover [40]. In contrast, daytime temperatures were not related to impervious surface and had a weaker, negative relationship with tree cover. In Madison, Wisconsin (USA), spatial patterns of daytime air temperature were negatively associated with tree cover summarized at spatial scales of 60–90 m [75]. A study of the urban heat island in Beijing, China found that the positive association between land surface temperature and impervious surfaces became stronger as these variables were summarized in larger spatial windows from 30 m up to 960 m [76], and an analysis of urban heat islands in three cities in Southeast Asia found a similar result [77]. Buildings, pavement, and other impervious surfaces absorb large amounts of solar energy during the daytime and release this energy at night, resulting in a broad-scale urban heat island effect on minimum temperatures. In contrast, tree cover has more localized effects on maximum temperature as a result of shading and evapotranspiration during the daytime.

In addition to their microclimate effects, land use and land cover influence mosquito population dynamics and disease transmission through a variety of other pathways. Spatial variation of vegetation, topography, and the built environment provides diverse breeding and resting habitats for mosquitoes, which in turn influence mosquito abundance and potential exposure of human and zoonotic hosts to questing mosquitoes. For container-breeding mosquitoes the abundance and quality of artificial habitats is particularly important and has been found to vary considerably across urban landscapes [37, 78–80]. Neighborhood-level variation in socio-economic factors, such as housing conditions, sanitation, and accessibility of public health resources, also influence the types and abundances of artificial breeding habitats as well as human exposure to mosquitoes and the overall risk of disease transmission [26, 27].

This study considered only thermal effects on vectorial capacity and was not intended to provide a comprehensive assessment of dengue transmission risk. However, our modeling results demonstrated that different thermal regimes are an important proximal effect of land use and land cover on mosquito habitat and mosquito-borne disease transmission cycles. In Athens-Clarke County GA, variation in microclimate temperatures was sufficient to result in a doubling of vectorial capacity along a rural-to-urban gradient. We expect these effects to be even greater in larger metropolitan areas such as Atlanta, where urban heat island effects are stronger than in smaller cities. Therefore, we recommend that efforts at mapping the risk of mosquito-borne disease transmission in heterogeneous urban landscapes should consider the effects of microclimate variation as a potential driver of disease transmission in addition to other factors influencing habitat and exposure to mosquito bites.

Spatio-temporal models of mosquito abundance and mosquito-borne disease transmission typically treat land cover as having a fixed, constant effect [33, 34]. However, our results showed that land cover effects on thermal suitability for mosquito-borne disease transmission exhibit seasonal change. These changes arise because of the non-linear mechanistic relationships between vectorial capacity and temperature. In early- to mid-summer, maximum temperatures regularly exceeded the optimum level for mosquito survival, reducing mosquito abundance and vectorial capacity. During this part of the season, increasing tree cover reduced maximum temperature to more optimal levels and therefore had a strong positive association with vectorial capacity. In late summer and autumn, minimum temperatures decreased below the optimum level for mosquito abundance and for critical traits affecting disease transmission such as the extrinsic incubation rate, but remained higher in locations where high cover of impervious surfaces produced an urban heat island effect. As a result, the influence of percent impervious surface cover on vectorial capacity increased relative to the effects of tree cover in late summer and early fall. More generally, we expect that in situations where land cover has a strong influence on microclimate, the resulting influences of land cover on temperature-dependent vectorial capacity will vary seasonally depending on the climatic setting and the thermal sensitivities of the mosquito and pathogen.

The health benefits of urban greenspace are widely recognized and include reductions in some atmospheric pollutants [81], cooling effects that can ameliorate the health impacts of heat waves [82], provision of opportunities for outdoor physical activity [83], and a variety of positive psychological benefits [84]. However, our findings demonstrate that areas of greenspace also have the potential to serve as foci for infectious disease transmission. A study of *Ae*. *albopictus* abundance in Rome, Italy similarly found that the highest abundances was associated with “green islands” of vegetation located in heavily urbanized areas [85]. Forest edges in urban and suburban areas have been identified as important locations for tick-borne disease spillover into the human population [86]. In the Atlanta metropolitan area, forested areas with streams affected by combined sewer overflow were associated with high rates of human West Nile virus infection [87]. In these examples, urban forests provide favorable habitat for vectors and zoonotic hosts combined with opportunities for human exposure. Our results indicate that urban greenspace can also facilitate mosquito-borne disease transmission by providing microclimate refugia that enhance vectorial capacity. To optimize public health in urban environments, it will be essential to maximize the potential health benefits of urban greenspace while mitigating its potential negative impacts on vector-borne disease transmission.

Additional research and development of analytical tools and data products will be necessary to facilitate broader use of microclimate information to support mosquito-borne disease surveillance and control efforts in cities. The availability of inexpensive temperature loggers and free access to many sources of satellite remote sensing and climate data can facilitate the implementation of similar microclimate modeling studies in other locations. Depending on the geographic setting and the amount of data available, future studies should explore the sensitivity of microclimate to other important features such as water bodies, terrain, and soil characteristics. For more comprehensive risk assessments, predictions of thermal suitability for disease transmission will need to be combined with data on breeding habitat availability, human exposure, and other important ecological and epidemiological factors. Ultimately, it will be necessary to develop and validate generalizable models of urban mosquito microclimates and mosquito abundance that can be applied across multiple cities within a region to highlight where climate conditions are currently suitable for disease transmission and assess potential sensitivities to climate change.

## Supporting information

S1 File Supplementary methods for urban land cover mapping. (DOCX)

S2 File Supplementary results for mosquito abundance modeling, references, and Figures A-E.
(DOCX)
