# Supplementary material for: Land cover affects microclimate and temperature suitability for arbovirus transmission in an urban landscape
Source: PLoS Negl Trop Dis. 2020 Sep 21;14(9):e0008614. doi: 10.1371/journal.pntd.0008614 (PMC7529312; doi:10.1371/journal.pntd.0008614)
Supplement: S1 File — (DOCX) [file pntd.0008614.s001.docx]

Supporting Information: S1 Appendix

Land cover affects microclimate and temperature suitability for arbovirus transmission in an urban landscape

Michael C. Wimberly^1^*, Justin K. Davis^1^, Michelle V. Evans^2,3^, Andrea Hess^1^, Philip M. Newberry^2^, Nicole Solano-Asamoah^2,3^, Courtney C. Murdock^2,3,4,5,6,7, 8^*

^1^ Department of Geography and Environmental Suitability, University of Oklahoma, Norman OK, USA

^2^ Odum School of Ecology, University of Georgia, Athens, GA, USA

^3^ Center for Ecology of Infectious Diseases, University of Georgia, Athens, GA USA

^4^ Department of Infectious Diseases, University of Georgia, Athens, GA, USA

^5^ Center for Tropical Global and Emerging Diseases, University of Georgia, Athens, GA USA

^6^ Center for Vaccines and Immunology, University of Georgia, Athens, GA USA

^7^ River Basin Center, University of Georgia, Athens, GA USA

^8^Department of Entomology, College of Agriculture and Life Sciences, Cornell University, Ithaca, NY, USA

* Corresponding authors

Michael C. Wimberly ([mcwimberly@ou.edu](mailto:mcwimberly@ou.edu))

Courtney C. Murdock ([ccm256@cornell.edu](mailto:ccm256@cornell.edu))

**This PDF File Includes:**

Supplementary Methods

References

# Supplementary Methods

We mapped tree cover and impervious surfaces for Athens with satellite remote sensing using Sentinel-1 synthetic aperture radar (SAR) C-band Level-1 Ground Range Detected imagery combined with Sentinel-2 level 1-C top of atmosphere multispectral reflectance data. Land cover was mapped for 2017 because this was the most recent year for which very-high-resolution imagery were available to develop a training dataset. Visual examination of satellite imagery from 2017 and 2018 indicated that there was minimal change over this one-year period, and we were confident that the 2017 land cover map was suitable for modeling the 2018 microclimate data.

The Sentinel-1 data had a spatial resolution of 10 m for the vertically transmitted and received SAR backscatter signal (VV) and the vertically transmitted, horizontally received SAR backscatter signal (VH). We used a total of 23 Sentinel-1 images that were acquired between May 1, 2017 and September 30, 2017. The VV and VH bands were used to compute the normalized difference polarization index, which is sensitive to surface roughness and vegetation structure and can discriminate between bare and forested surfaces (1).

The Sentinel-2 data had a spatial resolution of 10 m for the visible and near-infrared bands and 20 m for the shortwave infrared bands. We used a total of 26 Sentinel-2 images that were acquired between May 1, 2017 and September 30, 2017, including 19 with less than 60% cloud cover and 13 with less than 20% cloud cover. Cloud-obscured pixels were screened using the cloud mask band provided with the data. These images were used to compute a variety of spectral indices, including the normalized difference vegetation index (2), the normalized difference water index (3), the normalized difference moisture index (4), the normalized burn ratio (5), and the normalized difference built-up index (6).

We randomly selected 350 training plots within the study area. Each square plot was 20 x 20 m and consisted of a 5 x 5 grid of sample points. The land cover type at each of these points was classified as 1) tree, 2) other vegetation, 3) impervious surface, 4) water, or 5) bare soil. These point-level estimates were then used to calculate percent tree cover and percent impervious surface for each plot. Random forest models were used to estimate the manually-interpreted tree cover and impervious surface as a function of the remotely sensed variables, which included median composites of all raw bands and derived indices from Sentinel-1 and Sentinel-2. The random forest algorithm is a machine learning technique that uses an ensemble of decision or regression trees to predict the dependent variable based on hierarchical series of binary splits in the predictor variables (7). The algorithm creates multiple trees, in our case, 1000 trees for each classification. For each tree, a random subset of the observations is sampled with replacement. For each split within the tree, a subset of predictor variables is randomly sampled without replacement. The final random forests classification output for a given set of predictor variables is based on the mean of the predictions from all the component trees. We carried out image processing and random forest modeling using Google Earth Engine (8).

These models were applied to predict 2017 tree cover and impervious surface across the entire study area. The resulting maps had relatively high accuracies, with mean absolute error (MAE) of 12.9% and a predicted-observed correlation of 0.87 for tree cover, and MAE of 9.1% and a predicted-observed correlation of 0.86 for impervious surface based on the out-of-bag predictions.

# References

1. Hird J, DeLancey E, McDermid G, Kariyeva J. Google Earth Engine, open-access satellite data, and machine learning in support of large-area probabilistic wetland mapping. Remote Sens. 2017;9(12):1315.

2. Pettorelli N, Vik JO, Mysterud A, Gaillard J-M, Tucker CJ, Stenseth NC. Using the satellite-derived NDVI to assess ecological responses to environmental change. Trends Ecol Evol. 2005;20(9):503-10.

3. McFeeters SK. The use of the Normalized Difference Water Index (NDWI) in the delineation of open water features. Int J Remote Sens. 1996;17(7):1425-32.

4. Gao B-C. NDWI—A normalized difference water index for remote sensing of vegetation liquid water from space. Remote Sens Environ. 1996;58(3):257-66.

5. García ML, Caselles V. Mapping burns and natural reforestation using Thematic Mapper data. Geocarto Int. 1991;6(1):31-7.

6. Zha Y, Gao J, Ni S. Use of normalized difference built-up index in automatically mapping urban areas from TM imagery. Int J Remote Sens. 2003;24(3):583-94.

7. Belgiu M, Drăguţ L. Random forest in remote sensing: A review of applications and future directions. ISPRS J Photogramm Remote Sens. 2016;114:24-31.

8. Gorelick N, Hancher M, Dixon M, Ilyushchenko S, Thau D, Moore R. Google Earth Engine: Planetary-scale geospatial analysis for everyone. Remote Sens Environ. 2017;202:18-27.
