# Supplementary material for: Land cover affects microclimate and temperature suitability for arbovirus transmission in an urban landscape
Source: PLoS Negl Trop Dis. 2020 Sep 21;14(9):e0008614. doi: 10.1371/journal.pntd.0008614 (PMC7529312; doi:10.1371/journal.pntd.0008614)
Supplement: S2 File — Supplementary results for mosquito abundance modeling, references, and Figures A-E. (DOCX) [file pntd.0008614.s002.docx]

Supporting Information: S2 Text

Land cover affects microclimate and temperature suitability for arbovirus transmission in an urban landscape

Michael C. Wimberly^1^*, Justin K. Davis^1^, Michelle V. Evans^2,3^, Andrea Hess^1^, Philip M. Newberry^2^, Nicole Solano-Asamoah^2,3^, Courtney C. Murdock^2,3,4,5,6,7,8^*

^1^ Department of Geography and Environmental Suitability, University of Oklahoma, Norman OK, USA

^2^ Odum School of Ecology, University of Georgia, Athens, GA, USA

^3^ Center for Ecology of Infectious Diseases, University of Georgia, Athens, GA USA

^4^ Department of Infectious Diseases, University of Georgia, Athens, GA, USA

^5^ Center for Tropical Global and Emerging Diseases, University of Georgia, Athens, GA USA

^6^ Center for Vaccines and Immunology, University of Georgia, Athens, GA USA

^7^ River Basin Center, University of Georgia, Athens, GA USA

^8^Department of Entomology, College of Agriculture and Life Sciences, Cornell University, Ithaca, NY, USA

* Corresponding authors

Michael C. Wimberly ([mcwimberly@ou.edu](mailto:mcwimberly@ou.edu))

Courtney C. Murdock ([ccm256@cornell.edu](mailto:ccm256@cornell.edu))

**This PDF File Includes:**

Supplementary Results

Supplementary Figure A-J

# Supplementary Results

## Monthly patterns of minimum and maximum temperature

Minimum temperatures were highest in the center of Athens-Clarke County where there was high cover of impervious surfaces and lowest in the outlying rural areas (Fig A in S2 Text). In contrast, maximum temperatures were highest in areas of low tree cover, which included developed areas in urbanized parts of the county as well as fields and other clearings in the rural portions of the county (Fig B in S2 Text). Maximum temperatures were highest in June-July, decreased slightly in July-August, increased in August-September, and then decreased again in September-October. Minimum temperatures were highest in June-July and decreased continuously throughout the season.

## Empirical and Mechanistic Mosquito Abundance Predictions

Surface plots of monthly *M(T)* predictions as a function of monthly mean minimum and maximum temperatures illustrated the different temperature sensitivities of the empirical model based on microclimate and mosquito data collected in the field and the mechanistic model based on temperature-trait relationships from laboratory experiments. The empirical model predicted the highest *M*(*T_min_, T_max_*) at warm minimum (20-22 °C) and cool maximum (29-31 °C) temperatures (Fig C in S2 Text). The mechanistic model predicted the highest *M(T)* at cooler minimum (19.5-20.5 °C) and maximum (< 30 °C) temperatures (Fig D in S2 Text). The abundances predicted by the mechanistic model were also much higher than those predicted by the empirical model. These higher values are understandable because the mechanistic model uses a simple equilibrium population equation based on parameters derived under idealized laboratory conditions and does not consider ecological interactions such as competition, predation, and habitat availability that have large influences on mosquito population growth and carrying capacity.

The differences were less pronounced for the *VC(T)* models, which shared the same equations and parameters for the disease transmission components of the model. However, predictions of *VC(T)* based on empirical estimates *of M*(*T_min_, T_max_*) were still highest at warm minimum (> 21.0 °C) and relatively cool maximum (30-31.5 °C) temperatures (Fig E in S2 Text). In contrast, the *VC(T)* predictions based on theoretical *M(T)* estimates peaked at slightly cooler minimum (20.5-21.5 °C) and maximum (29.5-30.5 °C) temperatures (Fig F in S2 Text). Because of the differences in the magnitude of the mosquito abundance predictions, the predictions of *VC(T)* based on the mechanistic *M(T)* estimates were much higher than those based on the empirical *M*(*T_min_, T_max_*) estimates.

In Athens-Clarke County, the warmest minimum temperatures were concentrated in the urbanized core where there was high cover of impervious surfaces (Fig A in S2 Text), and the coolest maximum temperatures occurred in patches of high tree cover in the urban core as well as the surrounding rural areas (Fig B in S2 Text). As a result, the highest mosquito abundances predicted by the empirical *M*(*T_min_, T_max_*) model occurred in patches of trees embedded within the more developed central portion of the county (Fig G in S2 Text). Predicted abundance increased from June-July to a peak in July-August and then decreased into late summer and early fall. In contrast, the mechanistic *M(T)* model predicted higher mosquito abundances in outlying rural areas than in the urban core, and in the fall than in the summer (Fig. H in S2 Text). These differences in mosquito abundance predictions also led to differences in predictions of *VC(T)* that incorporated the empirical estimates of mosquito abundance (Fig. I in S2 Text) versus mechanistic estimates of mosquito abundance (Fig. J in S2 Text).


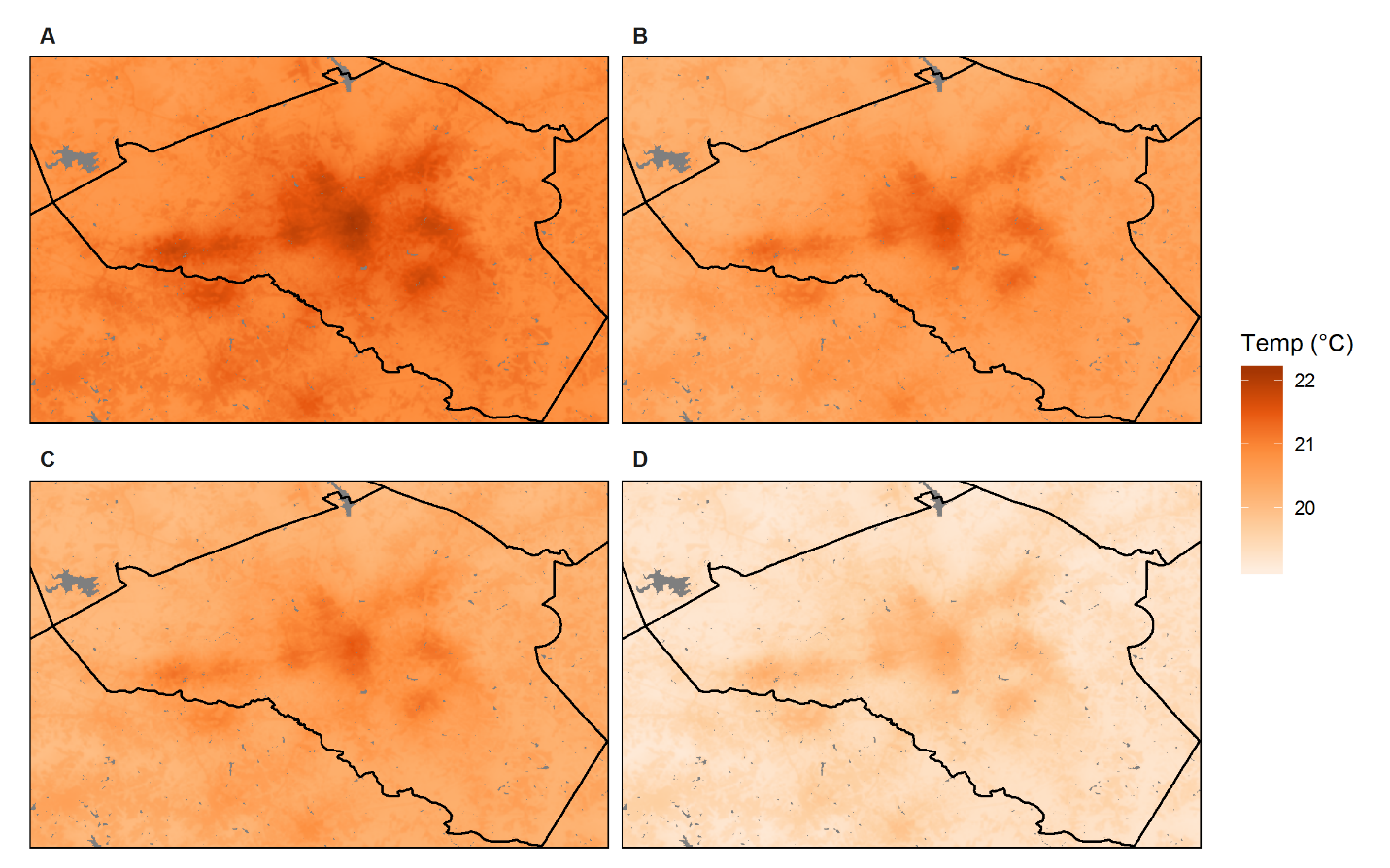


**Figure A**: Mean minimum microclimate temperatures summarized during four monthly periods in 2018. A) June-July. B) July-August. C) August-September. D) September-October. The maps were produced using R version 3.6.1. The maps were produced using R version 3.6.1.


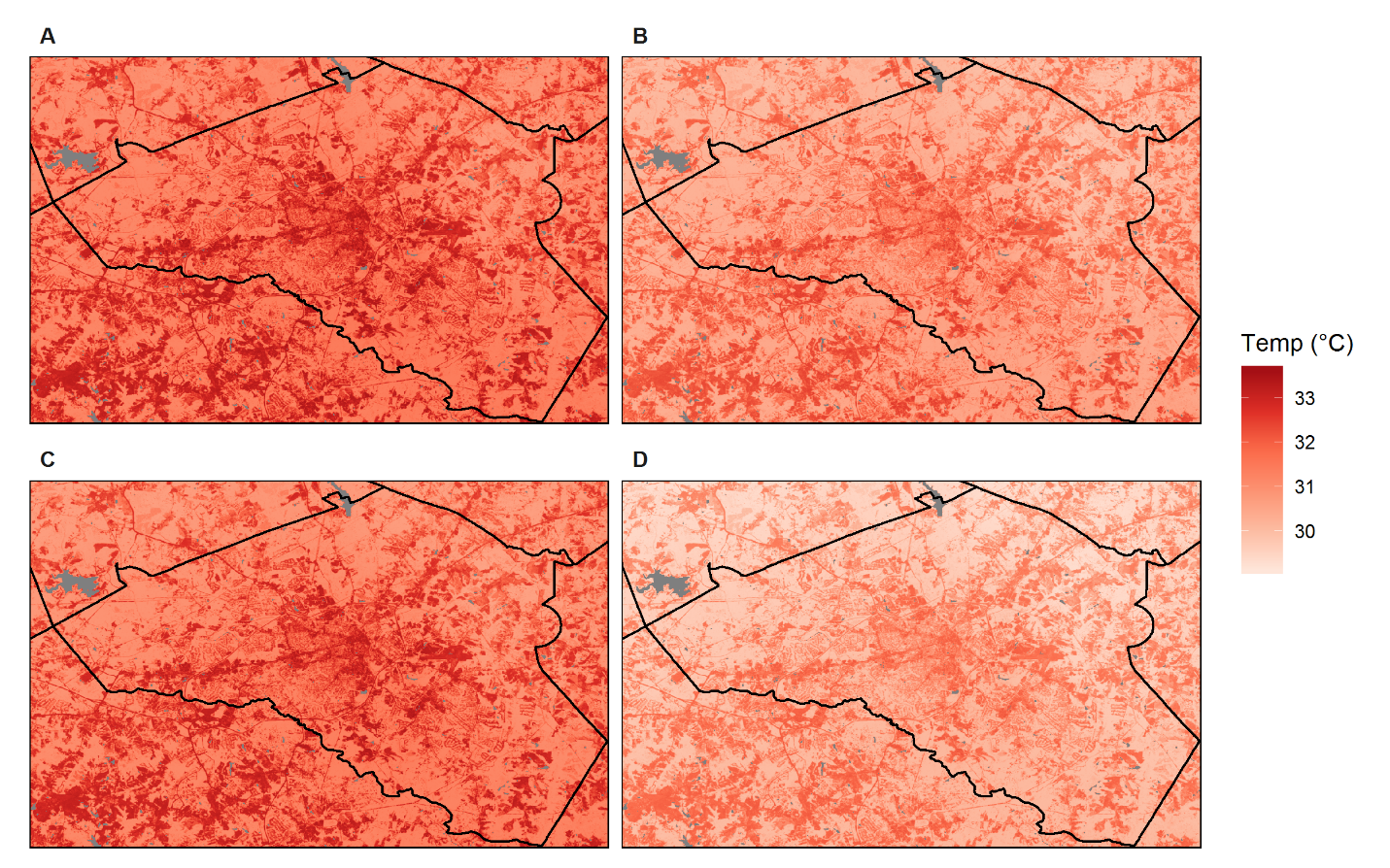


**Figure B**: Mean maximum microclimate temperatures summarized during four monthly periods in 2018. A) June-July. B) July-August. C) August-September. D) September-October. The maps were produced using R version 3.6.1. The maps were produced using R version 3.6.1.


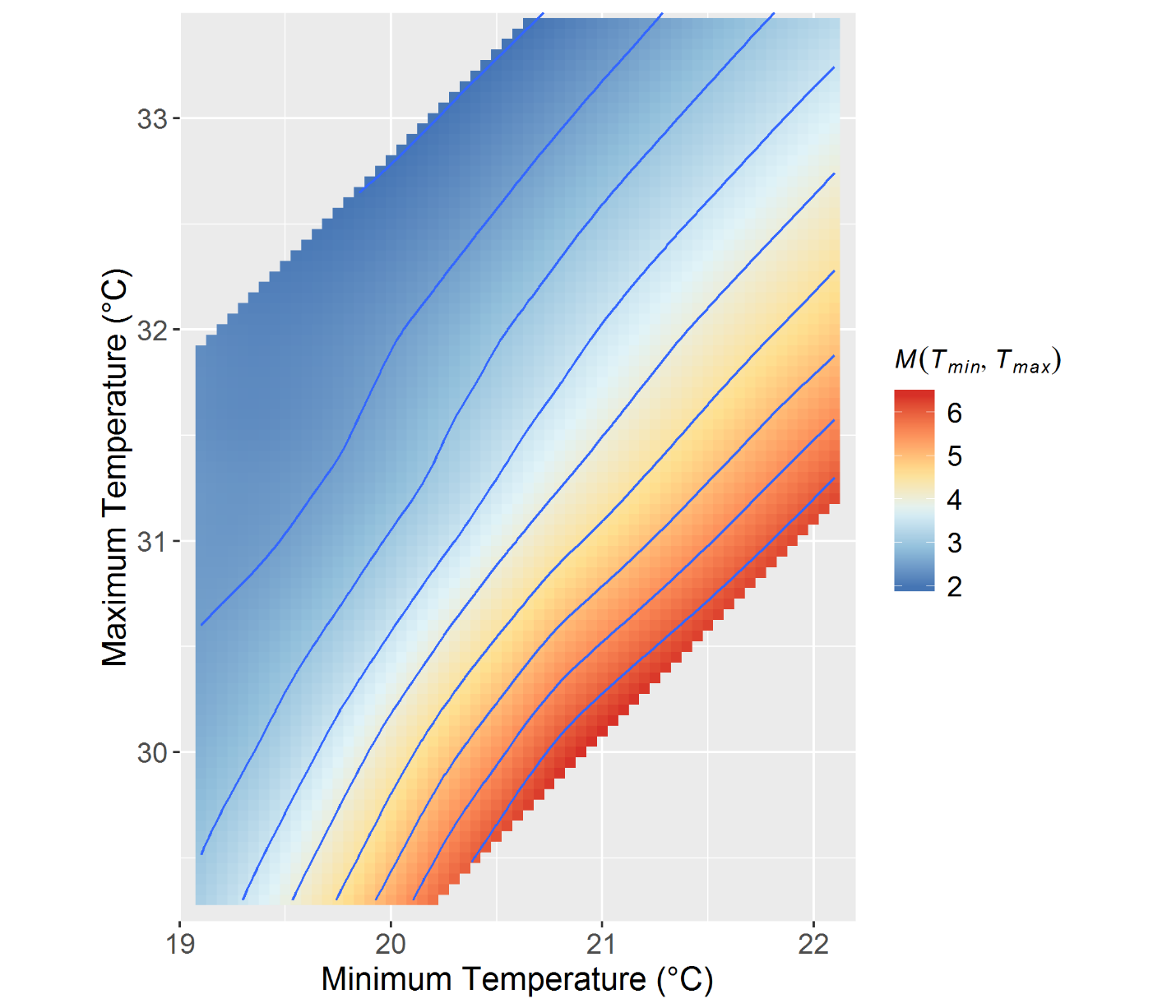


**Figure C**: Contour plot of monthly mosquito abundance predicted by the empirical *M(T_min_, T_max_)* model in relation to mean minimum and maximum microclimate temperatures. The response surface was generated using locally estimated scatterplot smoothing (LOESS) regression. Each blue contour line represents a change in daily mosquito abundance of 0.5.


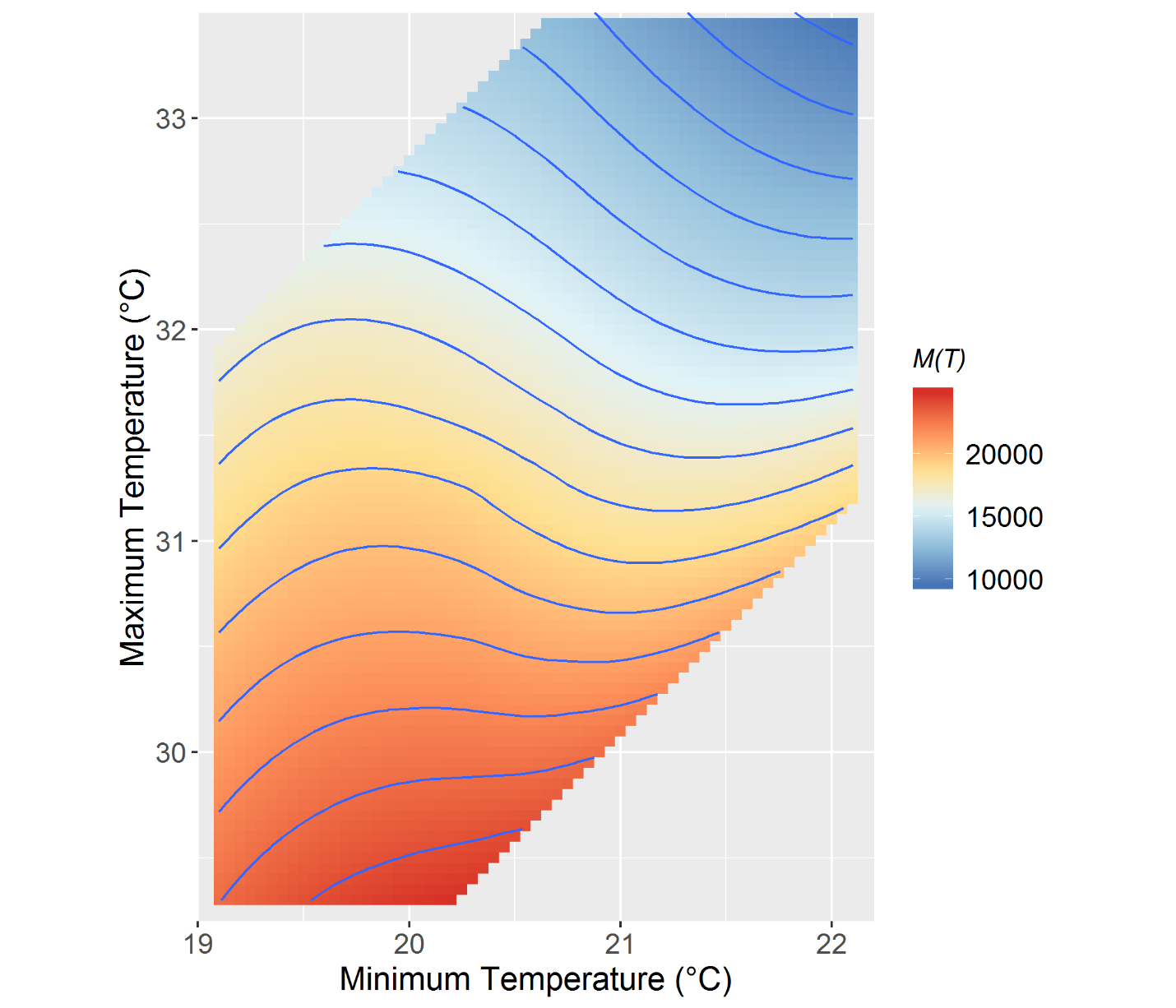


**Figure D**: Contour plot of monthly mosquito abundance predicted by the mechanistic *M(T)* model in relation to mean minimum and maximum microclimate temperatures. The response surface was generated using locally estimated scatterplot smoothing (LOESS) regression. Each blue contour line represents a change in daily mosquito abundance of 1000.


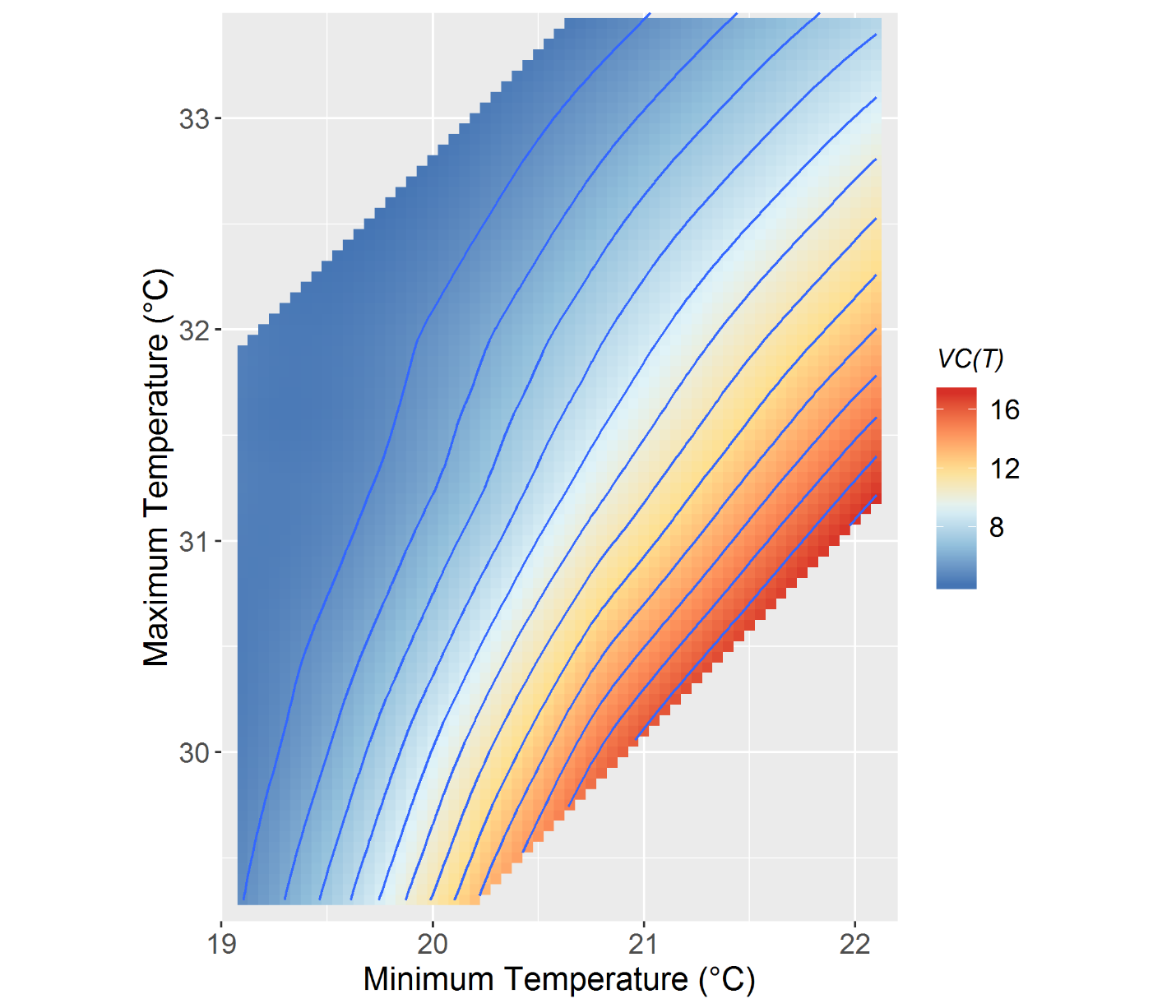


**Figure E**: Contour plot of monthly vectorial capacity predictions based on empirical *M(T_min_, T_max_)* estimates in relation to mean minimum and maximum microclimate temperatures. The response surface was generated using locally estimated scatterplot smoothing (LOESS) regression. Each blue contour line represents a change in vectorial capacity of 1.


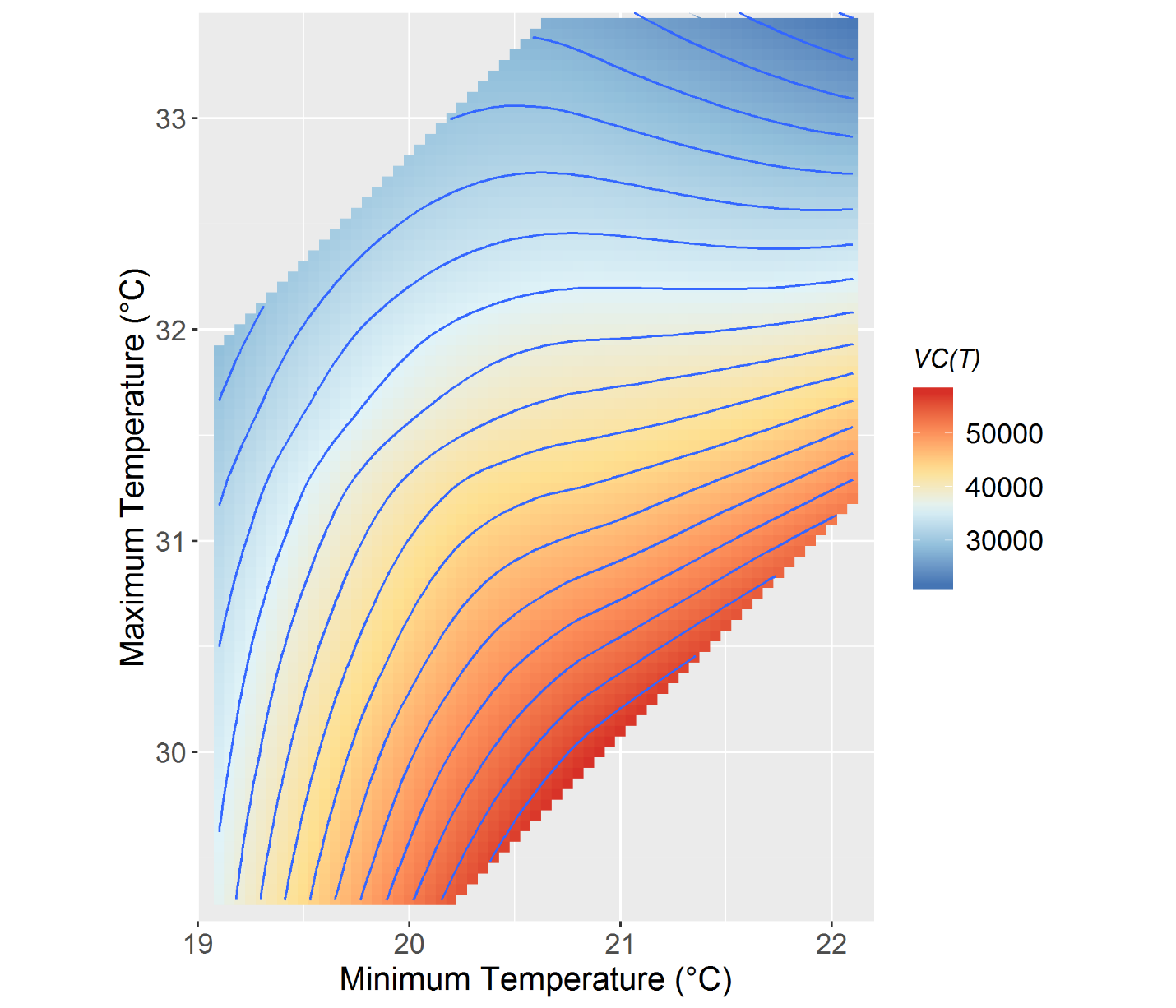


**Figure F**: Contour plot of monthly vectorial capacity predictions based on mechanistic *M(T)* estimates in relation to mean minimum and maximum microclimate temperatures. The response surface was generated using locally estimated scatterplot smoothing (LOESS) regression. Each blue contour line represents a change in vectorial capacity of 2000.


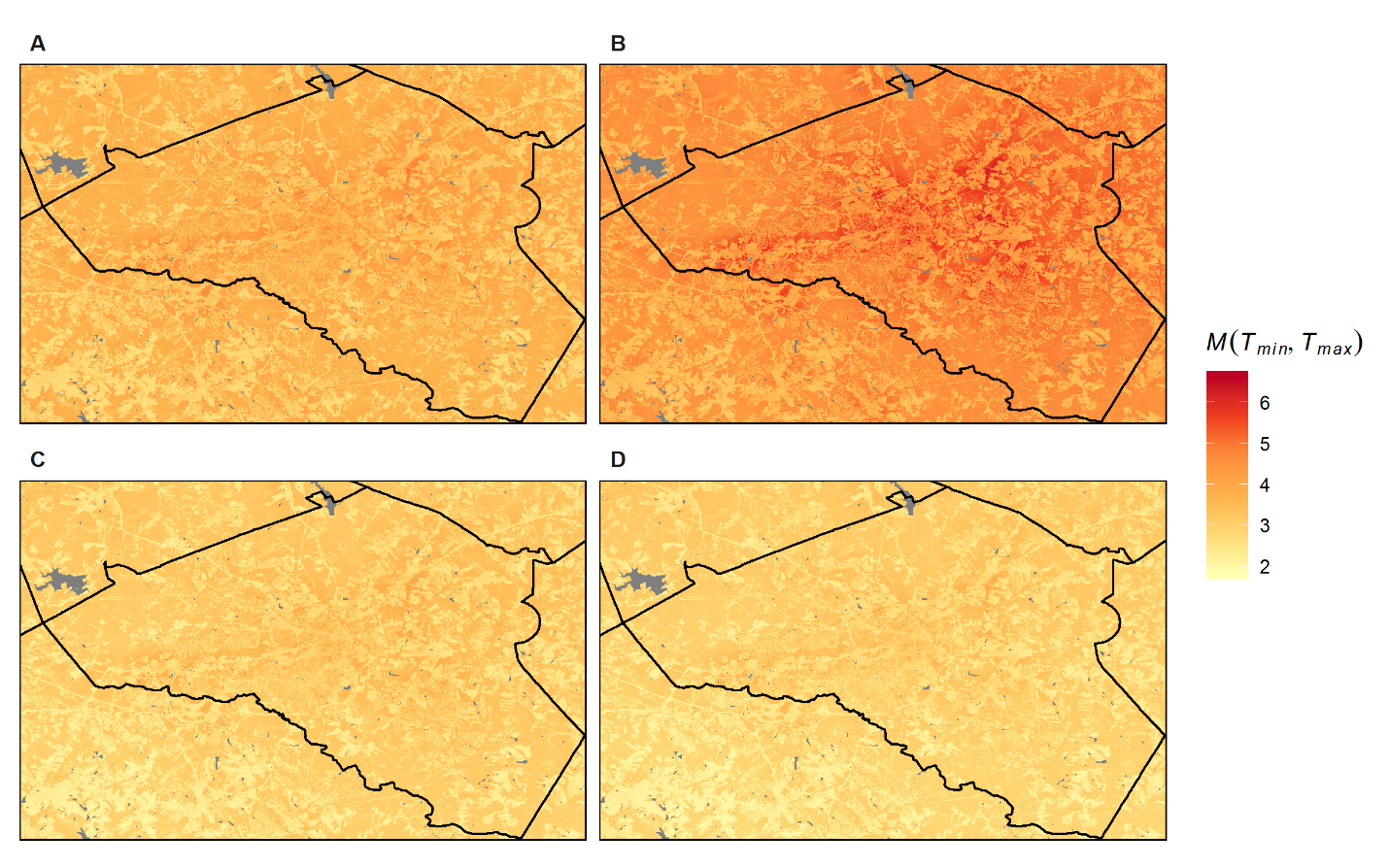


**Figure G**: Predicted densities of *Ae. albopictus* mosquitoes during four monthly periods in 2018. A) June-July. B) July-August. C) August-September. D) September-October. The maps were generated by combining the empirical *M(T_min_, T_max_)* model illustrated in Fig. C with daily microclimate data. The maps were produced using R version 3.6.1.


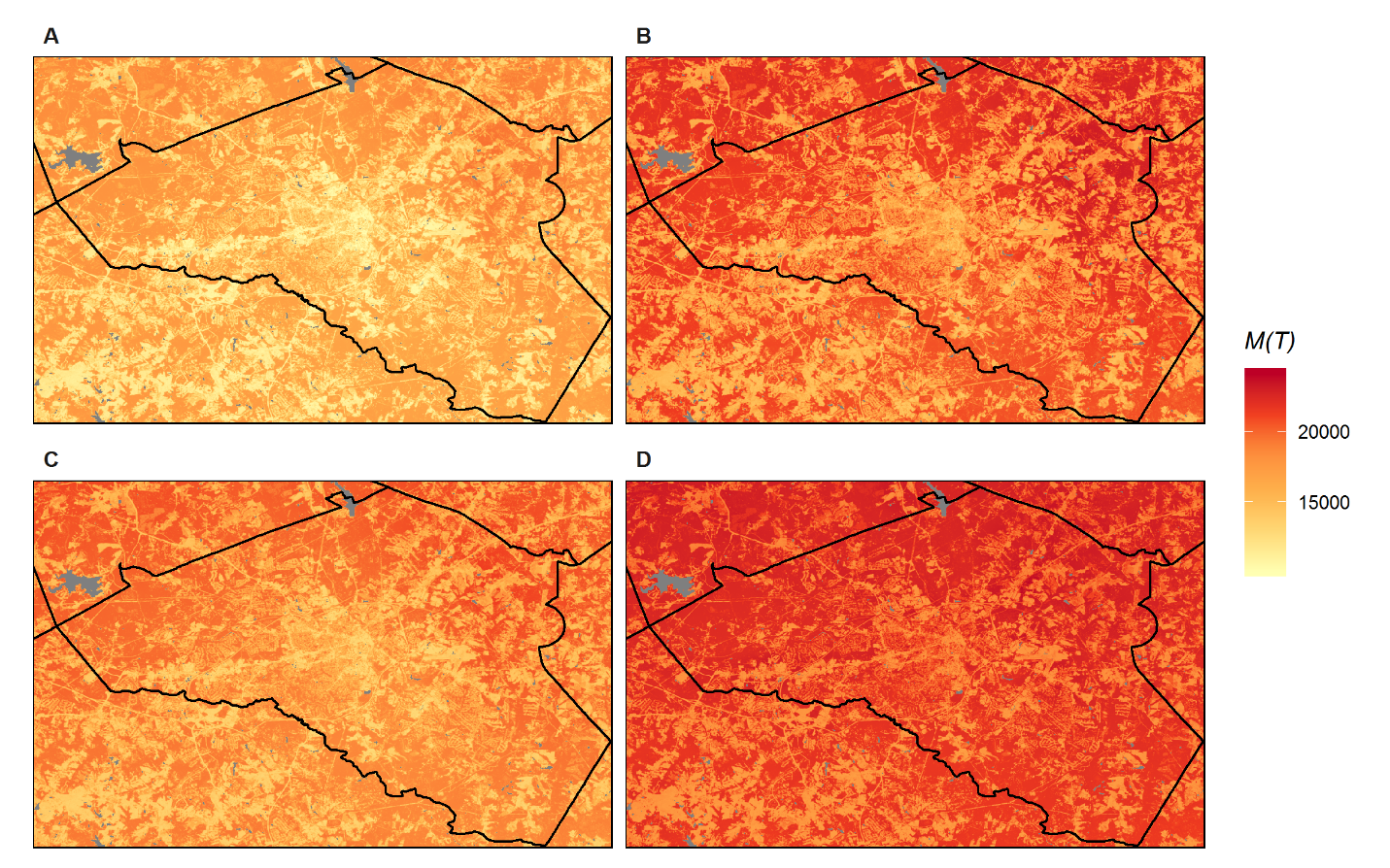


**Figure H**: Predicted densities of *Ae. albopictus* mosquitoes during four monthly periods in 2018. A) June-July. B) July-August. C) August-September. D) September-October. The maps were generated by combining the mechanistic *M(T)* model with daily microclimate maps. The maps were produced using R version 3.6.1.


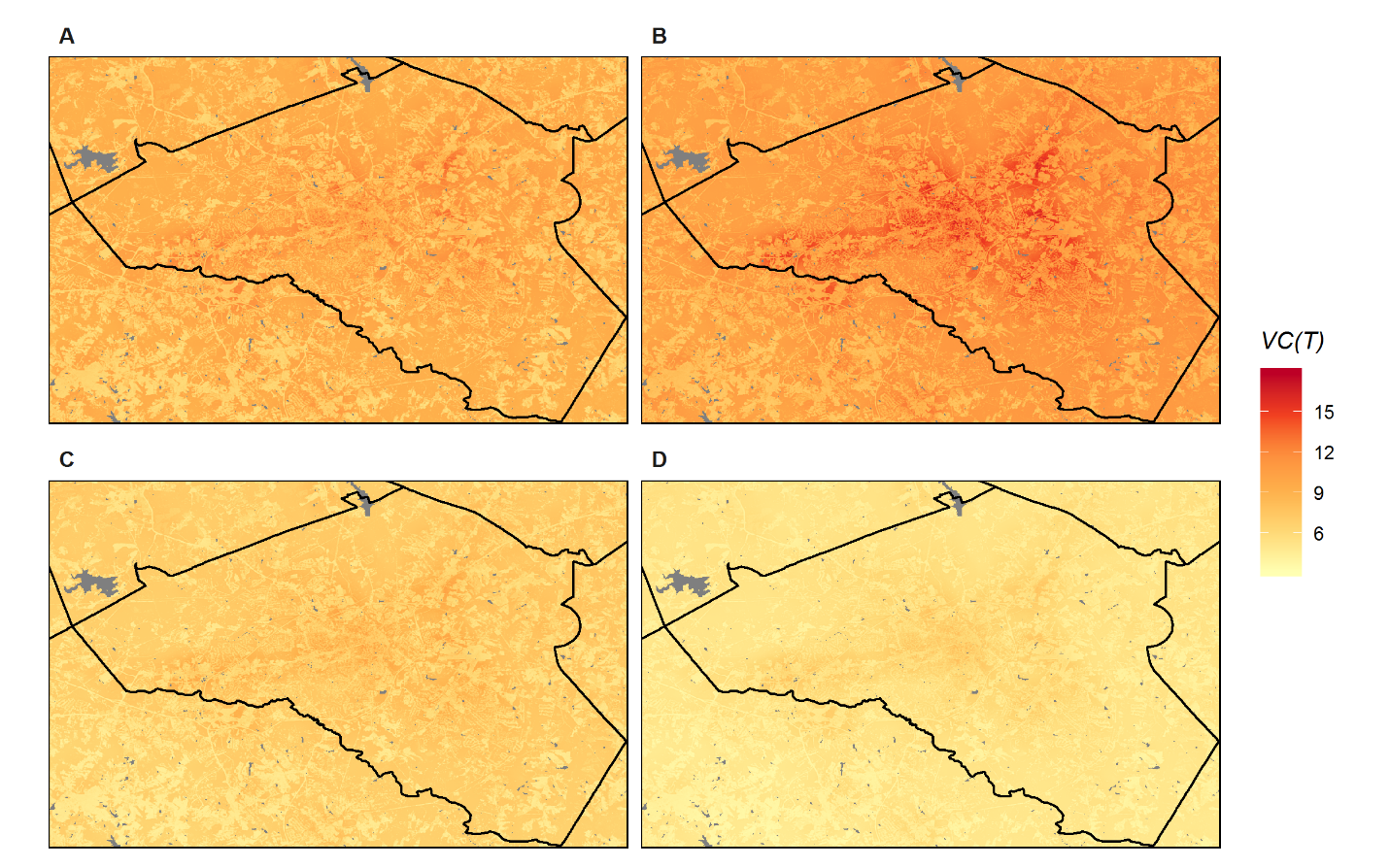


**Figure I**: Predicted vectorial capacity during four monthly periods in 2018. A) June-July. B) July-August. C) August-September. D) September-October. The maps were generated by combining the empirical model of mosquito abundance with the temperature-trait model of vectorial capacity. The maps were produced using R version 3.6.1.


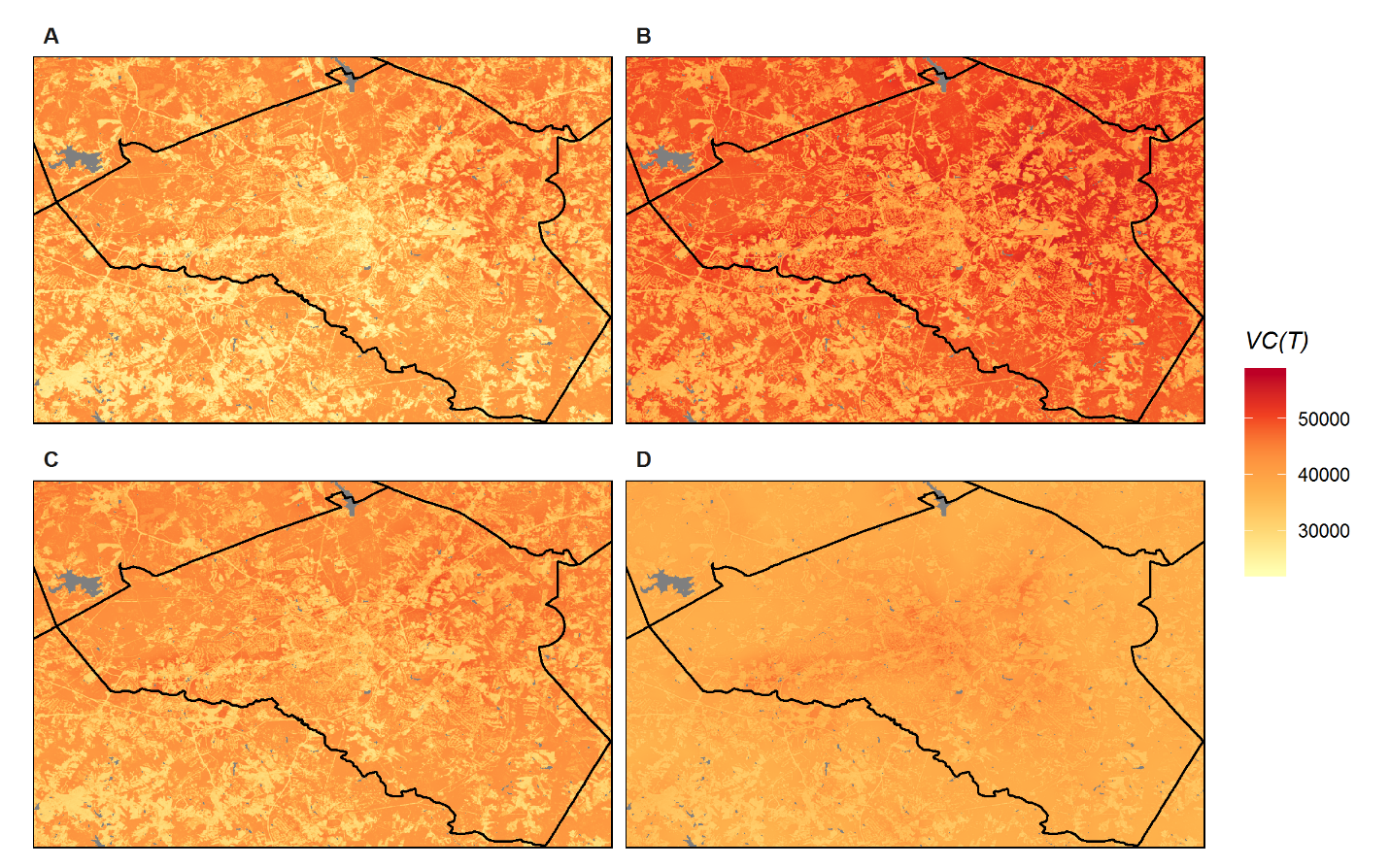


**Figure J**: Predicted vectorial capacity during four monthly periods in 2018. A) June-July. B) July-August. C) August-September. D) September-October. The maps were generated by combining the mechanistic model of mosquito density with the temperature-trait model of vectorial capacity. The maps were produced using R version 3.6.1.
